# Molecular and Functional Interactions Between Cisplatin and Nicotinamide: A Combined Computational, Spectroscopic, and Biological Study

**DOI:** 10.3390/ijms27114989

**Published:** 2026-05-30

**Authors:** Beata Szefler, Magdalena Wujak, Agnieszka Skotnicka, Krzysztof Skowron, Julia Czuba, Przemysław Czeleń, Kamil Szupryczyński, Piotr Cysewski

**Affiliations:** 1 Department of Physical Chemistry, Faculty of Pharmacy, Collegium Medicum, Nicolaus Copernicus University, Kurpińskiego 5, 85-096 Bydgoszcz, Poland; przemekcz@cm.umk.pl (P.C.); piotrc@cm.umk.pl (P.C.); 2 Department of Medicinal Chemistry, Faculty of Pharmacy, Collegium Medicum in Bydgoszcz, Nicolaus Copernicus University, Jurasza 2, 85-089 Bydgoszcz, Poland; mwujak@cm.umk.pl; 3 Faculty of Chemical Technology and Engineering, Bydgoszcz University of Science and Technology, Seminaryjna 3, 85-326 Bydgoszcz, Poland; askot@pbs.edu.pl; 4 Department of Microbiology, Faculty of Pharmacy, Collegium Medicum in Bydgoszcz, Nicolaus Copernicus University, M. Curie Skłodowskiej 9, 85-094 Bydgoszcz, Poland; krzysztof.skowron@cm.umk.pl (K.S.); julia.czuba2001@wp.pl (J.C.); 5 Faculty of Pharmacy, Doctoral School of Medical and Health Sciences, Collegium Medicum, Nicolaus Copernicus University, Jagiellońska 13, 85-067 Bydgoszcz, Poland; 503592@doktorant.umk.pl

**Keywords:** cisplatin, nicotinamide, lung cancer, anticancer activity, antimicrobial activity, molecular modelling

## Abstract

Cisplatin remains a widely used anticancer agent; however, its effectiveness can be influenced by systemic toxicity, resistance mechanisms, and interactions with exogenous compounds. Nicotinamide (vitamin B3), an NAD^+^ precursor and a commonly used dietary supplement, is involved in cellular metabolism, redox homeostasis, and DNA repair pathways, which may potentially modulate the cellular responses to Platinum-based agents. Here, we combine chemical synthesis, computational studies, spectroscopic analysis, and biological assays to investigate the molecular and biological aspects of Cisplatin–Nicotinamide interactions. A novel *cis*-[Pt(NH_3_)_2_NicotinamideCl]NO_3_ complex was obtained and its structure analyzed. Density functional theory (DFT) calculations indicate a thermodynamically favorable coordination of Nicotinamide to the first hydrolysis product of Cisplatin (CisPt1) with binding energies comparable to those calculated for nucleobase coordination under the same theoretical conditions. In non-small cell lung cancer cell lines (A549 and PC-9), in vitro results suggest that Nicotinamide pre-treatment reduces Cisplatin cytotoxicity under specific experimental conditions, but the pre-formed complex does not exert anticancer effects. These data are consistent with a model in which Nicotinamide may interact with reactive Cisplatin species, potentially contributing to the reduced availability of reactive Platinum(II) species. This work provides mechanistic insight into potential drug–nutrient interactions involving Platinum-based chemotherapy and highlights the need for further investigation under clinically relevant conditions in the near future.

## 1. Introduction

### 1.1. Overview of Cisplatin: Mechanism of Action, Side Effects, and Antimicrobial Potential

Cisplatin (CisPt, CDDP), is the most widely used of the Platinum-containing chemotherapy drugs and continues to be a key agent in the treatment of a variety of solid tumors, including those of the lung, ovary, testis, bladder, head, and neck [1,2,3,4,5,6,7,8,9]. Since its introduction in 1968, Cisplatin has been employed in both monotherapy and combination regimens [6,10,11,12,13,14,15].

**Figure 1:**
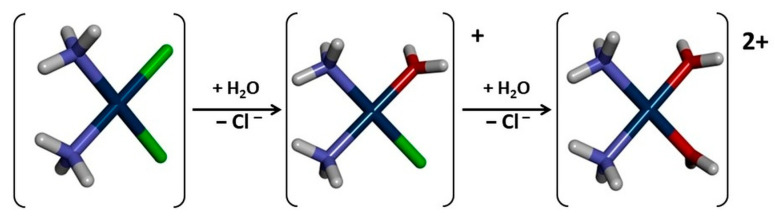

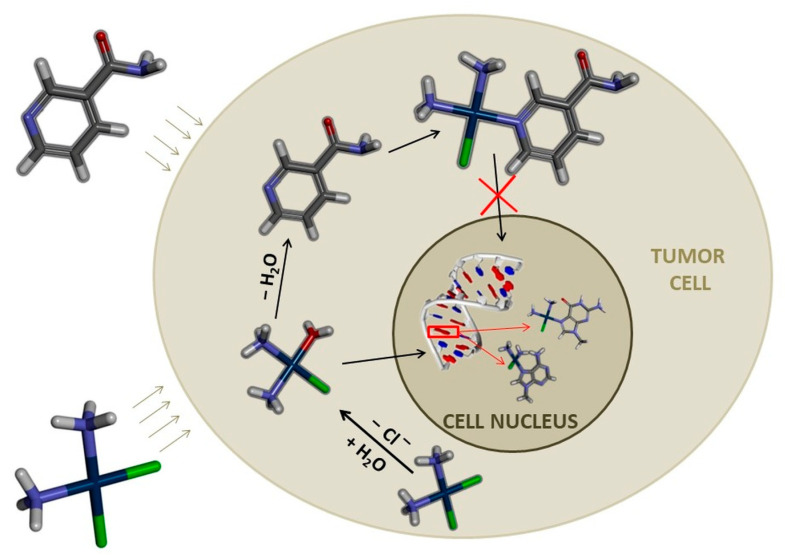
Scheme of the first and second steps of the Cisplatin (CisPt) hydrolysis in the cytoplasm of a tumor [15] with the anticancer activity pathway of the first product of hydrolysis (CisPt1) of Cisplatin (CisPt) and the coordination tendencies of Nicotinamide (B3) for nucleobases in DNA, specifically Adenine (A) and Guanine (G).

Cisplatin undergoes hydrolysis inside the cell, at the molecular level, and produces highly reactive aquated species that bind to nucleophilic positions within DNA, mostly at N7 of Guanine and Adenine bases, to form DNA crosslinks that cause apoptosis and transcriptional stalling (Figure 1) [16]. Additionally, Cisplatin induces reactive oxygen species (ROS), further enhancing its cytotoxicity [4]. Nevertheless, the pharmacological destiny of Cisplatin is not specified by DNA-binding alone. Still, the clinical applicability of Cisplatin therapy is undermined by considerable systemic toxicity, a small therapeutic window, side effects that limit dosage, and the walls of intrinsic and acquired resistance mechanisms. Cisplatin carries “black box warnings” for nephrotoxicity (kidney damage) [17,18], peripheral neuropathy (nerve damage) [19,20,21], severe nausea and vomiting [22,23], and myelosuppression (bone marrow suppression, leading to increased infection risk) in clinical guidelines [24,25,26]. Other serious adverse effects include ototoxicity (hearing loss) [27,28] and arrhythmias, including bradycardia and tachycardia [29,30]. The lack of selectivity of Cisplatin, which also affects healthy, rapidly dividing cells, contributes significantly to these side effects [3,4,31]. The persistent challenge of high systemic toxicity means that the therapeutic window for Cisplatin is narrow [32]. This inherent limitation underscores the necessity for strategies that either reduce the effective dose or enable targeted delivery to minimize adverse effects [33,34,35,36]. This has prompted intense investigations of the molecular mechanisms regulating Cisplatin activity, transport, activation, and inactivation within living systems. Recent evidence suggests that both intracellular and extracellular molecules of biological origin are capable of modifying Cisplatin reactivity, distribution, and availability. Simultaneously, there has been a dramatic expansion in dietary supplement use among cancer patients. For example, Nicotinamide (vitamin B3) is a widely used dietary and therapeutic supplement in metabolic and skin diseases [37,38,39,40,41,42]. Nicotinamide, a precursor of NAD^+^, is essential for cellular metabolism, oxidative stress management, DNA repair, and epigenetic regulation. Based on these biological functions, Nicotinamide may influence chemotherapeutic response.

### 1.2. Role of Vitamin B3 (Nicotinamide): Biological Functions and Interaction Potential

Vitamin B3, in particular its amide form Nicotinamide, is a water-soluble vitamin with a wide range of biological functions [37,38,39,40,41,42]. It is a metabolic precursor of NAD^+^ (Nicotinamide Adenine dinucleotide), a coenzyme necessary for ATP generation, cellular metabolism, DNA synthesis and repair, and many other processes [43]. Recent studies suggest direct antimicrobial effects of Nicotinamide and its potential to boost host defense via stimulating production of antimicrobial peptides (AMPs) in the skin [41]. The antimicrobial mechanisms of action involve microbial cell cycle arrest, disorder, and dispersal of the DNA coupled with direct interaction and penetration into the DNA that results in DNA damage, which avoids efficient replication [44]. Nicotinamide is also a PARP-1 (poly-ADP-ribose polymerase-1) inhibitor, an enzyme that takes part in DNA repair [44,45]. Research indicates that vitamins such as B3 can potentiate antibiotics for efficacy against MDR bacteria, possibly by altering bacterial metabolism or by enhancing the immune response [46].

Nicotinamide was deliberately selected for this study due to its significance in biology as a vitamin and as a possible N-donor ligand. The pyridine nitrogen of Nicotinamide offers in medicinal chemistry a distinct coordination position which can affect the electrophilicity of the Platinum center and, hence, its reactivity towards genomic DNA. Such molecular interactions might alter the drug behavior in a biological milieu, and investigation is certainly warranted. In silico (molecular docking) [36,47,48] and spectroscopic (UV-Vis) [36,49,50,51,52,53,54] studies have previously suggested significant interactions between Platinum-based drugs (Cisplatin, Oxaliplatin, Carboplatin) and B vitamins, indicating affinity and potential for complex formation. Critically, B vitamins share structural similarities with DNA nucleobases, possessing aromatic rings with lone pairs of electrons, which could contribute to potential drug–nutrient coordination tendencies and potentially impact their therapeutic efficacy [51,52,53,54]. Nicotinamide’s multifaceted biological roles, including its direct and indirect antimicrobial properties and structural resemblance to DNA nucleobases, make it an intriguing candidate for the study [44]. The structural similarity to DNA nucleobases and its role in DNA repair pathways (as a PARP-1 inhibitor and NAD^+^ precursor) present a complex scenario [55]. This is consistent with the possibility that Nicotinamide either undergoes coordinate covalent bonding with Cisplatin species, thereby antagonizing its cytotoxicity, or alternatively, influences cellular processes in a favorable manner. This dual (synergistic/antagonistic) nature of the interaction should be defined in detail to know exactly what to expect in terms of effects in both anticancer and antimicrobial applications. The clinical importance of these interactions is further underlined by investigations of the potential for the binding affinity of Cisplatin to B vitamins to interfere with therapeutic efficacy in patients who take in B vitamin-rich foods. This highlights the need to monitor consumption of dietary or supplemental B vitamins whilst undergoing chemotherapy.

### 1.3. Antimicrobial Activity of Cisplatin

In addition to its anticancer effect, Cisplatin has a somewhat underexploited record in antimicrobial efficacy. Its growth inhibitory effect on *Escherichia coli* was noted as far back as in 1967, even before that of its powerful anticancer activity [14]. The literature survey suggests that Cisplatin also possesses a wide spectrum of antimicrobial activity against both Gram-negative and Gram-positive bacteria and some strains of yeast and mold [56,57].

### 1.4. Rationale and Objectives

Although Nicotinamide metabolism is biologically relevant in cancer biology [36,49,50,51,52,53,54], potential direct molecular interactions of Nicotinamide with Platinum-based agents have not been explored systematically. Notably, no combined chemical, computational, and biological study has yet been reported on this interaction.

The current work has the ambition to (i) investigate the molecular interactions of Cisplatin with Nicotinamide, (ii) provide an evaluation of their thermodynamic and structural aspects based on computational chemistry methods, (iii) analyze the biological impact of these interactions in cancer cell models and assessment of antimicrobial effectiveness (since infectious complications remain a major cause of morbidity and mortality in oncology patients, any factor capable of modulating the antimicrobial properties of Cisplatin may be of potential clinical relevance), and (iv) suggest a mechanistic model of in vitro bioactivity modulation. By combining chemistry, computation, and cell biology, this study gives a multiscale framework for drug–nutrient interactions with Platinum-based chemotherapeutics.

## 2. Results

### 2.1. Chemistry

#### Characterization of the *cis*-[Pt(NH_3_)_2_NicotinamideCl]NO_3_ Complex

The structure of the synthesized complex was confirmed by analysis of the ^1^H and ^13^C NMR spectra. NMR spectra clearly indicate the presence of aromatic proton signals in the range of 7.68–9.13 ppm, as well as five distinct signals corresponding to the carbon atoms of the Nicotinamide aromatic ring, which can be seen at δ = 126.3–155.4 ppm. Furthermore, the presence of two N–H protons from the amide group of the Nicotinamide moiety was confirmed by signals at 8.37 and 7.90 ppm. The most characteristic carbon atom, i.e., the carbonyl carbon atom C-7, was observed at 164.7 ppm. It is imperative to emphasize that two discrete singlets were detected at 4.65 (3H) and 4.33 (3H) ppm, respectively, for the protons of the two ammonia ligands that were coordinated to the Platinum center. In *cis*-diaminePlatinum (II) complexes, the two ammonia ligands are chemically non-equivalent due to the overall asymmetry of the complex introduced by the presence of different ligands (Nicotinamide and chloride) in the coordination sphere. The observation of two distinct singlets for the ammonia protons provides strong and unambiguous evidence for the *cis* configuration of the synthesized complex. If the complex adopted a *trans* geometry, only one averaged signal for the equivalent ammonia protons would be observed. Confirmation of the *cis* geometry is paramount, as the stereochemistry of Platinum complexes significantly impacts their biological activity, including their ability to interact with DNA and their overall pharmacological profile.

The signals of the NH_3_ groups in the ^15^N NMR spectra of the *cis*-[Pt(NH_3_)_2_NicotinamideCl]NO_3_ complex can be seen at −422.9 and −414.8 ppm, respectively. These values are consistent with the nitrogen atoms of the two non-equivalent ammonia ligands. The distinctness of these peaks further corroborates the *cis* geometry of the complex, a finding that is consistent with the ^1^H NMR results. The signal at −270.5 ppm (N-8) was assigned to the nitrogen atom of the amide group (-CONH_2_), while the signal at −169.8 ppm (N-1) was assigned to the pyridine nitrogen, which is directly coordinated to the Platinum center. Nitrogen atoms directly coordinated to a heavy metal center like Platinum typically experience a significant downfield effect. In comparison, the chemical shift value for the uncomplexed pyridine nitrogen atom in Nicotinamide is only −64.5 ppm, and for the amide group nitrogen it is −277.1 ppm [58] (−270.5 ppm for the complex studied).

### 2.2. Computational Study

Our in vitro findings, which showed NSCLC cell lines as highly sensitive to Cisplatin, are consistent with the known mechanism of action [59]. It is a well-established conclusion in Cisplatin chemistry that mono-aquated Platinum species are the dominant thioester-forming electrophiles and that these species, rather than the diaqua species, react with DNA, accounting for more than 95% of its DNA adducts [60,61,62]. This process of DNA platination eventually leads to cell damage and cell death. The decrease in efficacy of Cisplatin observed in A549 and PC-9 cells after co-treatment with Nicotinamide could be explained by two not mutually exclusive mechanisms: Nicotinamide may interfere directly with the generation of DNA–Platinum adducts, or it may promote DNA repair. The latter is particularly important since Nicotinamide controls cellular NAD+ levels required by DNA repair enzymes.

#### Calculated Thermodynamic Properties

**Figure 2:**
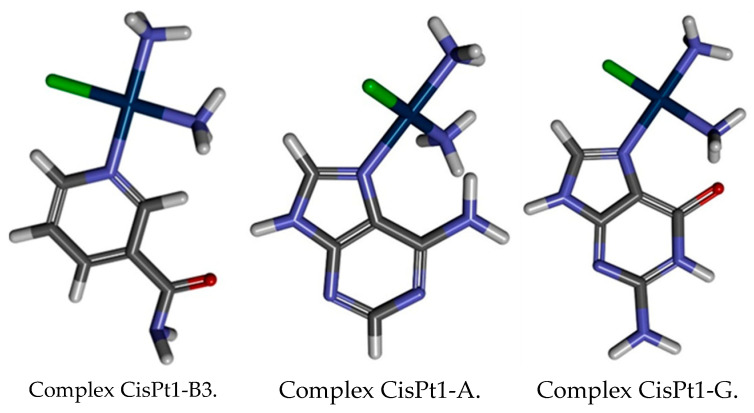
Optimized structures of *cis*-[Pt(NH_3_)_2_Cl(L)]^+^ complexes, where L represents Nicotinamide (B3) and nucleobases (Adenine (A), Guanine (G)), obtained at the MN15/def2-TZVP level of theory. Specifically, “Complex CisPt1–B3” refers to the *cis*-[Pt(NH_3_)_2_Cl(Nicotinamide)]^+^ complex.

To investigate the thermodynamic favorability of the interaction, the Gibbs Free Energy of reaction (ΔG_r_) for the complexation of Nicotinamide (B3) with the first hydrolysis product of Cisplatin (*cis*-[Pt(NH_3_)_2_Cl]^+^, or CisPt1) was determined. Calculations were performed at the B3LYP/6-31G(d,p)/LANL2DZ and MN15/def2-TZVP computational levels, with results summarized in Table 1 and illustrated in Figure 2.

In addition to the direct complexation with Nicotinamide, an essential point of this work was to compare the calculated thermodynamic parameters for Cisplatin and Nicotinamide complexation to those for the known Cisplatin–DNA nucleobase interactions with Adenine (A) and Guanine (G). This comparison is essential for evaluating the relative Cisplatin affinity for Nicotinamide over its major biological target in DNA and is detailed in Table 1. Evaluating these partial molecular interactions allows us to consider whether Nicotinamide has the potential to modulate Cisplatin’s binding to genetic material. The thermodynamic parameters thus provide a conceptual tool to judge possible interferences or synergies between the drug and B vitamins, both from the supplement and nutrient perspectives, at the molecular level.

### 2.3. Spectroscopy Results

#### 2.3.1. Theoretical UV-Vis Spectral Characteristics of Individual Components

Supplementary Figures S7 and S8 present the simulated UV-Vis absorbance spectra of the constituents of the investigated mixtures: Adenine (A), Guanine (G), Nicotinamide (B3), and Cisplatin (CisPt). These spectra are important, as they define what is the normal absorbance for each reagent prior to or at early stages of complex formation. This baseline information can be used to separate the background absorbance of the starting materials completely from any new or modified spectral features produced solely by the complexation.

Supplementary Material Figures S7 and S8 report the CisPt–Nicotinamide (CisPt1-B3) and CisPt–nucleobases (Adenine, Guanine) (CisPt1-A, CisPt1-G) adducts’ theoretical UV-Vis absorbance spectra. Quantum-mechanical calculations predict the existence of complexes between Cisplatin and Nicotinamide as well as with nucleobases such as Adenine and Guanine (Supplementary Material Figures S6 and S7).

#### 2.3.2. Experimental UV-Vis Study

To verify the results of in silico assessment, an experimental study was conducted by UV-Vis spectroscopy. All the UV-Vis spectra in this work were recorded in pH 7.4 phosphate buffer to simulate the physiological condition of the blood plasma [63]. It is worth mentioning that the tumor microenvironment is usually more acidic with pH values ranging from 6.5 to 6.8 [16]. Previous studies suggest that the pH-dependent hydrolysis and reactivity of the Platinum-based drugs change [64,65].

**Figure 3:**
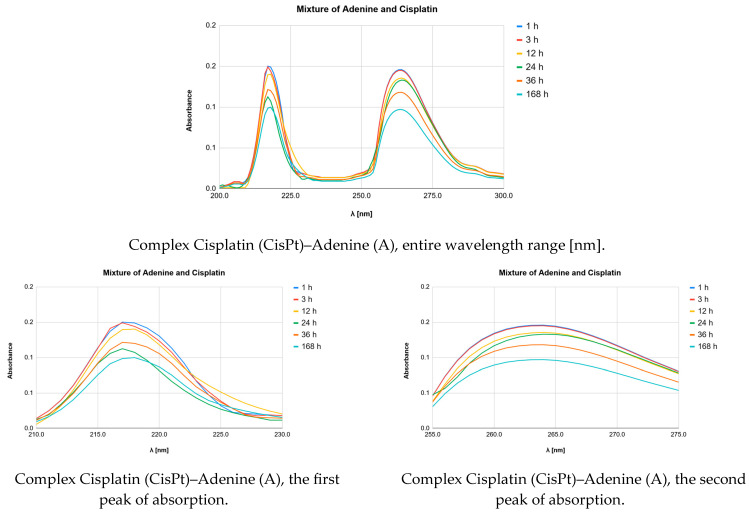

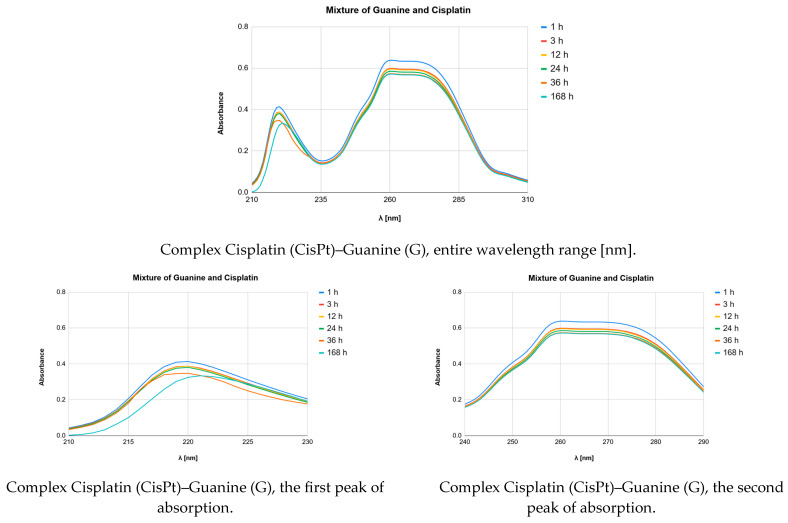
UV-Vis absorbance spectrum of a mixture of nucleobase (Adenine (A), Guanine(G)) and Cisplatin (CisPt, CDDP) during incubation at 37 °C in phosphate buffer at pH 7.4. Concentrations of 32.7 µM for Cisplatin and 65.3 µM for the nucleobases were maintained, representing a 1:2 ratio.

**Figure 4:**
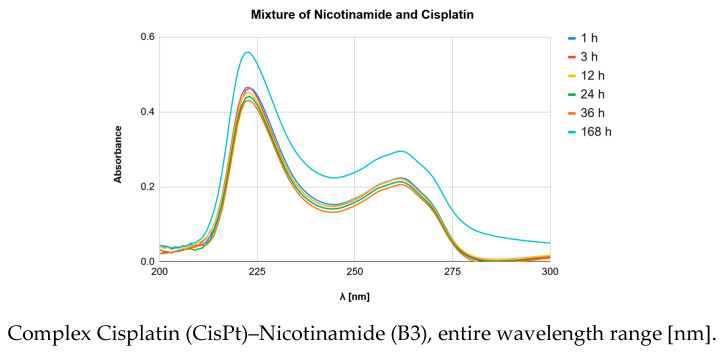

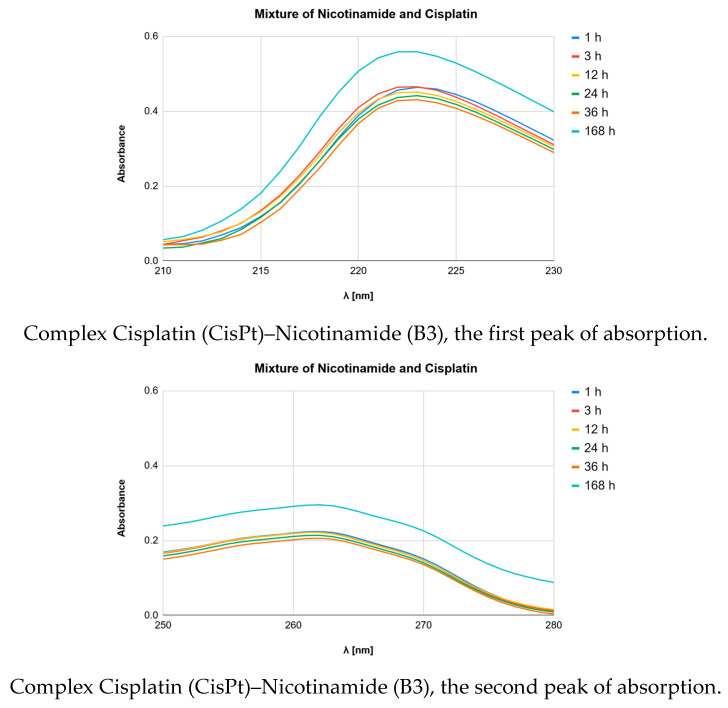
UV-Vis absorbance spectrum of a mixture of Nicotinamide (B3) and Cisplatin (CisPt, CDDP) during incubation at 37 °C in phosphate buffer at pH 7.4. Concentrations of 32.7 µM for Cisplatin and 65.3 µM for the Nicotinamide were maintained, representing a 1:2 ratio.

The complexes were studied within a 200 nm to 500 nm wavelength range and their UV-Vis spectra are shown in Figure 3 and Figure 4. The experimental spectra of Cisplatin–Nicotinamide complexes were compared with those of the Cisplatin complexes with the nucleobases Adenine (A) and Guanine (G) (Figure 3 and Figure 4). Further, spectra of pure Cisplatin (CisPt, CDDP), Nicotinamide (B3), and the corresponding nucleobases in phosphate buffer were recorded (Figure S6). The absorption maxima for all the pure sample fractions and mixtures were investigated.

**Figure 5:**
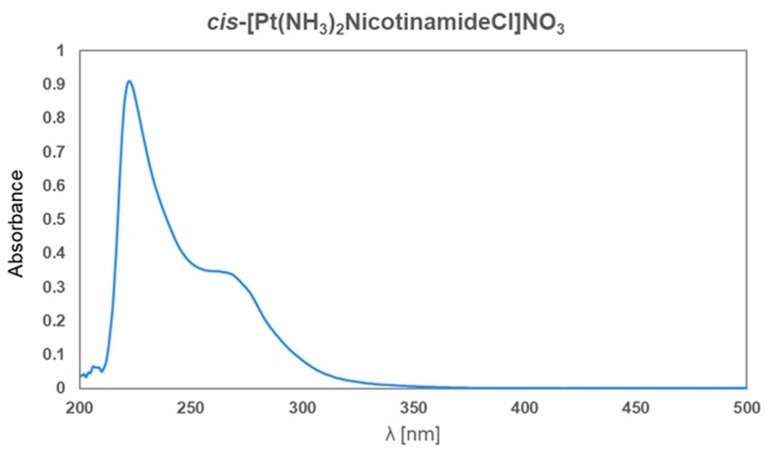
UV-Vis absorbance spectrum of a synthesized complex *cis*-[Pt(NH_3_)_2_NicotinamideCl]NO_3_ during incubation at 37 °C in phosphate buffer at pH 7.4.

The UV VIs spectrum of the complex *cis*-[Pt(NH_3_)_2_NicotinamideCl]NO_3_ was recorded (Figure 5).

#### 2.3.3. Theoretical UV-Vis Results and Comparison with Experiment

Table 2 shows a more detailed comparison of theoretically calculated UV-Vis absorption maxima (λ_max_) with the experimental ones for Nicotinamide (B3), Adenine (A), and Guanine (G) when complexed with Cisplatin as well as CisPt itself. Such a comparison is essential to ensure that the models are able to accurately predict the electronic features of these complexes. The MAD values between theory and experiment enable a quantitative evaluation of the agreement and the predictive quality of each method.

In the case of Adenine, the experimental results indicate two primary absorption bands at 218.0 nm and 264.0 nm. Both B3LYP and MN15 methods are consistent in predicting λ_max_ with the MADs being around 8–15 nm (MAD values of about 7–13 nm when calculated with respect to the absorbance values obtained from the literature data). The B3LYP method predicts the peaks at 237.2 nm and displays MADs resulting in 9.6 nm and 13.4 nm for the experimental bands (8.6 nm and 11.4 nm according to the literature data). MN15 gives similar results predicting values close to 234 nm with MADs of 8.0 nm and 15.0 nm (7.0 and 13.0 nm). These values of MAD imply that although the models represent the electronic transitions in the molecules rather well, small differences are present, and could be ascribed to solvent effects or to the incompleteness of basis sets for large molecules.

In the case of Guanine, experimental λ_max_ values are observed at 219.0 nm and 260.0 nm, without an additional shoulder around 249.0 nm suggested by the literature data. The B3LYP method predicts absorption at 232.5 nm, yielding MADs of 6.8 and 13.8 nm (6.8, 8.3, and 18.8 nm). The MN15 method provides similar predictions at 227.8 nm, with corresponding MADs of 4.4 and 16.1 nm (4.4, 10.6, and 21.1 nm). In the case of both levels of calculations, the MAD values for the second band in Adenine and Guanine are quite similar, at 13.4 nm and 13.8 nm, or 15.0 nm and 16.1 nm.

With regard to Nicotinamide (B3), the experimental absorption bands are at around 220 nm and 262 nm (no additional band at around 250 nm is observed as suggested by the literature data). B3LYP calculations give the λ_max_ of 246.4 nm for the proposed structure, which corresponds to the MADs of 13.2 and 7.8 nm (18.2, 1.8, or 6.8/7.8 nm). The predicted λ_max_ by the MN15 method is 244.6 nm, with MADs of 12.3 and 8.7 nm (17.3, 2.7, or 7.7/8.7 nm). It should be noticed that for Nicotinamide, the MAD values for MN15 are slightly smaller than those for B3LYP for the first band and slightly higher for the second band.

Finally, for Cisplatin (*cis*-[Pt(NH_3_)_2_Cl]^+^, (CisPt1)), the experimental absorption maxima are observed at 218 and 276 nm. The theoretical predictions for Cisplatin alone are crucial for establishing a baseline and understanding how coordination with ligands alters its electronic structure, and, consequently, its UV-Vis absorption. The general trend observed for the ligands indicates that the theoretical models are largely capable of reproducing the experimental UV-Vis profiles, albeit with varying degrees of accuracy depending on the specific molecule and the complexity of its electronic transitions. The MAD values highlight the inherent challenges in the precise modeling of complex charge transfer processes and ligand–metal interactions, particularly in solutions. Overall, the consistent, albeit sometimes disparate, results between the two computational methods and experimental data provide valuable insights into the electronic structure and stability of these biologically relevant Platinum complexes.

### 2.4. Biological Assays

The antimicrobial activity of Cisplatin, vitamin B3, and their mixtures varied significantly depending on the microbial strain and the method of mixture.

#### 2.4.1. Antimicrobial Activity of Cisplatin (CisPt)

The antimicrobial activity of Cisplatin varied with the strain. Gram-negative bacteria, *P. aeruginosa* (MIC 113 µM for both strains PAE ATCC 27853 and PAE1 (VIM-2)) and *E. coli* (MIC 450 µM for ECO ATCC 25922 and 275 µM for ECO 1 (ESBL+)), were the most susceptible to this drug. Against Gram-positive *S. aureus*, *E. faecalis*, *B. subtilis*, and *C. albicans*, the MIC CisPt values were exponentially higher. The MIC was 3600 µM for *S. aureus* (SAU ATCC 29213 and SAU1 (MRSA)). The MIC was 900 µM for EFA (*E. faecalis*) ATCC 29212 and 1800 µM for EFA1 (VRE). The MIC was 1800 µM for *B. subtilis* (BSU ATCC 7972 and BSU 1). For *C. albicans* (CAL ATCC 90028 and CAL 1 (fluconazole-resistant)), CisPt MIC was also 1800 µM. Surprisingly, *E. coli* ESBL+ and *K. pneumoniae* OXA-48 were more susceptible to CisPt alone (with lower MIC values) than their respective ATCC reference strains.

#### 2.4.2. Antimicrobial Activity of Vitamin B3 (B3)

In all tested strains, vitamin B3 showed no inhibition of microorganism growth across the entire range of tested concentrations, up to 3600 µM. The results tables consistently show the annotation MIC > 3600 (none), meaning that the MIC of vitamin B3 was higher than the highest tested concentration.

#### 2.4.3. Antimicrobial Activity of the Complex (Synthesized)

The activity of the synthesized CisPt1-B3 complex was mostly very low or non-existent. For most of the tested strains, no growth inhibition or an MIC at the highest tested concentration of 3600 µM was recorded. Exceptions were *P. aeruginosa* strains and *B. subtilis* strains, where the complex showed an MIC of 1800 µM. Compared to CisPt alone, the activity of the synthesized complex was generally significantly lower or equal to the highest concentration, suggesting that pre-forming the complex might have attenuated the antimicrobial activity of Cisplatin.

#### 2.4.4. Antimicrobial Activity of the CisPt + B3 Mixture (Fresh)

The following Table 3, Table 4 and Table 5 present detailed MIC results for individual substances and their mixtures, as well as a qualitative assessment of interactions. The freshly prepared CisPt+B3 (CisPt: B3 in 1:2 molar ratio) solution was active against different strains. MIC values of the mixture were 57/113 µM (for the PAE ATCC 27853) and 3600/7200 µM (for the EFA 1). Detailed MIC results for the single compounds and the mixtures are given in the following tables, as well as a qualitative estimation of the interactions.

### 2.5. Cell Line Study Results

The in vitro studies on NSCLC cell lines revealed complex interactions between Nicotinamide and Cisplatin.

#### 2.5.1. Effect of the Synthesized CisPt1-B3 Complex

The CisPt1-B3 complex (*cis*-[Pt(NH_3_)_2_NicotinamideCl]NO_3_ complex) prepared by chemical synthesis exhibited, as a general trend, significantly weaker cytotoxicity than Cisplatin in both cell lines (Table 6 and Table 7, Figures S11 and S12 in Supplementary Materials). In A549 cells, IC50 values of the CisPt1-B3 complex at all time points (24 h, 48 h and 72 h) were consistently higher than 125 µM, showing a dramatic loss of activity in comparison to Cisplatin with IC50 values of 50.86 ± 11.92 µM, 8.612 ± 1.402 µM, and 5.327 ± 0.733 µM, respectively. In the PC-9 cell line, the CisPt1-B3 complex showed IC50 values of >125 µM at 24 h, 84.19 ± 17.39 µM at 48 h and 29.85 ± 2.63 µM at 72 h. Although the complex exhibited cytotoxic activity at longer incubation times, its potency remained approximately tenfold lower than that of Cisplatin alone which presented IC50 values of 20.34 ± 6.276 µM, 2.107 ± 0.213 µM, and 0.990 ± 0.105 µM at the same time points. In addition, we evaluated the cytotoxicity of vitamin B3 alone and the freshly prepared mixture of CisPt and vitamin B3 (CisPt+B3 Mix, 1:2 ratio) on the two cell lines. No statistically significant difference was observed between the freshly prepared mixture of Cisplatin and vitamin B3, as compared to Cisplatin alone (Table 6 and Table 7). The results imply that the complexation between vitamin B3 and Cisplatin may have decreased the bioavailability or cellular efficacy of Cisplatin.

#### 2.5.2. Effect of Nicotinamide Pre-Incubation or Co-Treatment on Anticancer Cisplatin Activity

**Figure 6:**
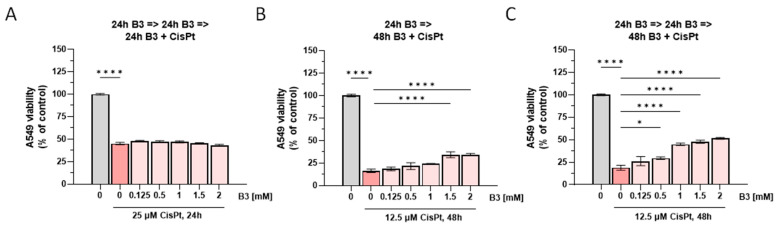
Effect of B3 on the Cisplatin (CisPt) anticancer activity in the A549 cell line. The vitamin was added every 24 h for in total 48 h (**A**), 24 h (**B**), and 48 h (**C**), followed by the final B3 addition in parallel with Cisplatin. Statistical significance was assessed using one-way ANOVA followed by Dunnett’s post hoc test, with each treatment group compared to the Cisplatin-only control. Significance levels are indicated on the figures as follows: * *p* < 0.05 and **** *p* < 0.0001. Statistical significance was assessed using one-way ANOVA followed by Dunnett’s post hoc test, with each treatment group compared to the Cisplatin-only control.

**Figure 7:**
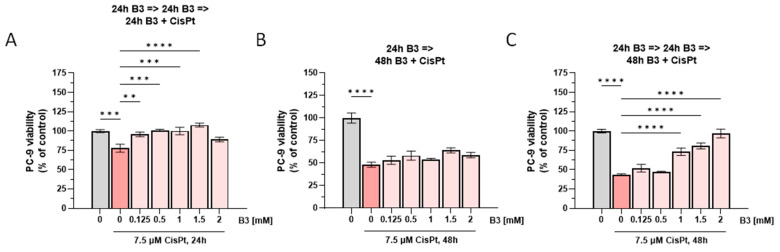
Effect of B3 on the Cisplatin (CisPt) anticancer activity in the PC-9 cell line. The vitamin was added every 24 h for in total 48 h (**A**), 24 h (**B**), and 48 h (**C**), followed by the final B3 addition in parallel with Cisplatin. Statistical significance was assessed using one-way ANOVA followed by Dunnett’s post hoc test, with each treatment group compared to the Cisplatin-only control. Significance levels are indicated on the figures as follows: ** *p* < 0.01, *** *p* < 0.001, and **** *p* < 0.0001.

In the case of B3 supplementation, every 24 h over a period of 24 or 48 h, followed by parallel B3 and CisPt addition for 24 h, no changes in the cytotoxic activity of the drug were observed for the A549 cell line (Figure 6A). Following 24 h pre-treatment with Nicotinamide (B3), and subsequent co-incubation with B3 and Cisplatin for 48 h, a partial reduction in Cisplatin cytotoxicity was observed at 1.5 mM and 2 mM concentration of B3, as evidenced by increased cell viability compared to Cisplatin alone (Figure 6B). Pre-treatment with B3 every 24 h over a 48 h period resulted in a more pronounced, dose-dependent reduction in the anticancer efficacy of Cisplatin (Figure 6C). In the PC-9 cell line, a single 24 h pre-treatment with Nicotinamide at concentrations up to 1.5 mM significantly increased cell viability compared to Cisplatin alone (Figure 7A). At the longer Cisplatin incubation time point (48 h), a non-significant trend toward increased cell viability was also observed following 24 h B3 pre-treatment (Figure 7B). In contrast, a pronounced and statistically significant reduction in Cisplatin anticancer activity was observed when PC-9 cells were pre-treated with B3 every 24 h for 48 h, followed by co-incubation with B3 and Cisplatin for 48 h. This effect was evident at B3 concentrations in the range of 1–2 mM (Figure 7C).

This partial of Cisplatin’s effect by higher Nicotinamide concentrations in NSCLC cell lines is a significant concern, suggesting that B vitamin supplementation could influence treatment efficacy.

**Figure 8:**
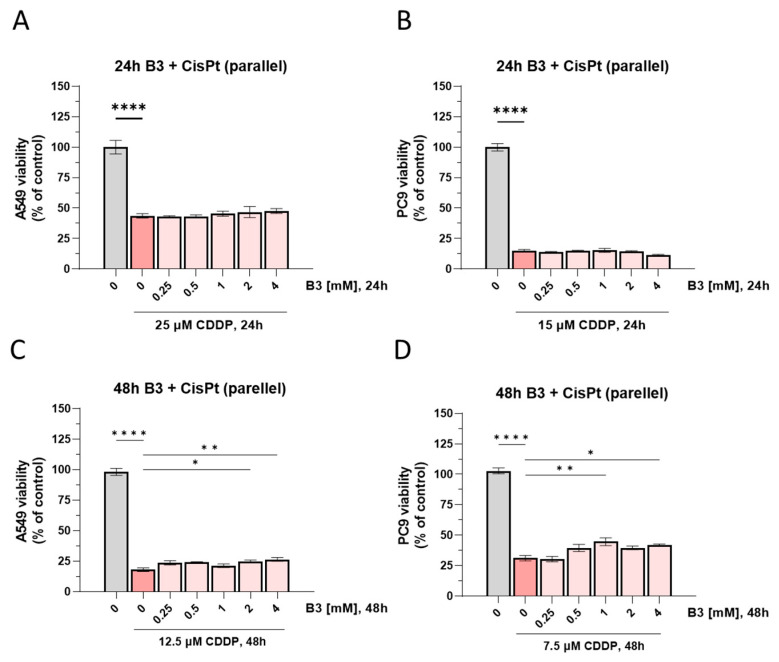
Effect of Nicotinamide (B3) on the Cisplatin (CisPt) anticancer activity in the A549 (**A**,**C**) and PC-9 (**B**,**D**) cell lines. Vitamin B3 and CisPt were administered concurrently for 24 h (**A**,**B**) and 48 h (**C**,**D**). Statistical significance was assessed using one-way ANOVA followed by Dunnett’s post hoc test, with each treatment group compared to the Cisplatin-only control. Significance levels are indicated on the figures as follows: * *p* < 0.05, ** *p* < 0.01, and **** *p* < 0.0001.

In the co-treatment with vitamin B3 and Cisplatin at high doses, for Cisplatin (0.25–4 mM; Figure 8) no differences in cytotoxic activity after 24 h were detected between the Cisplatin alone group (control) and any of the co-treatment groups (Figure 8A,B). After 48 h of co-incubation, a slight decrease in Cisplatin activity was observed at higher B3 concentrations for both cell lines, A549 and PC-9 (Figure 8C,D).

**Figure 9:**
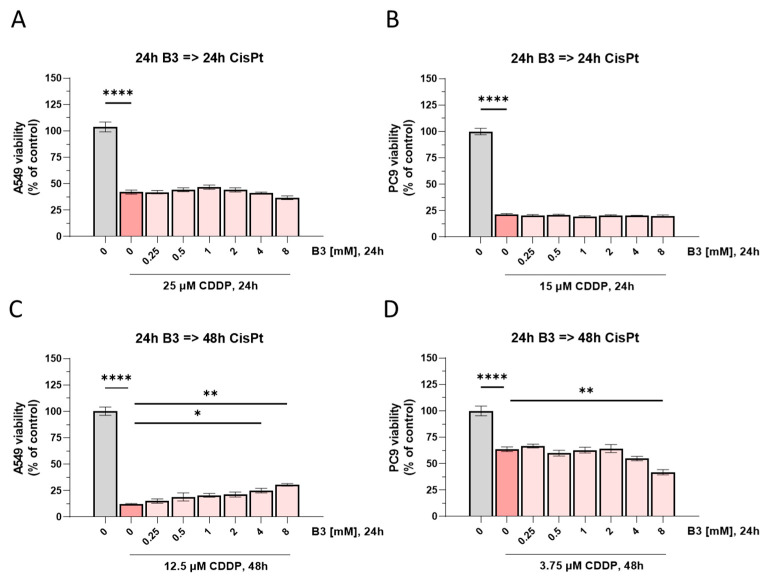
Effect of Nicotinamide (B3) on the Cisplatin (CisPt) anticancer activity in the A549 (**A**,**C**) and PC-9 (**B**,**D**) cell lines. The vitamin was added for 24 h, followed by the cell treatment with Cisplatin for 24 h (**A**,**B**) or 48 h (**C**,**D**). Statistical significance was assessed using one-way ANOVA followed by Dunnett’s post hoc test, with each treatment group compared to the Cisplatin-only control. Significance levels are indicated on the figures as follows: * *p* < 0.05, ** *p* < 0.01, and **** *p* < 0.0001.

In the sequential treatment experiment, cells were pre-treated with vitamin B3 for 24 h, followed by incubation with Cisplatin (i.e., without further B3 supplementation). As with the co-treatment experiment, 24 h treatment with Cisplatin did not result in any statistically significant changes in Cisplatin cytotoxicity in either A549 or PC-9 cells (Figure 9). After 48 h of Cisplatin treatment, a slight reduction in Cisplatin cytotoxicity was observed in A549 cells (Figure 9C), suggesting a partial antagonistic effect at certain B3 concentrations. In contrast, PC-9 cells did not exhibit this trend. At the highest B3 concentration (8 mM), co-treatment with Cisplatin resulted in decreased cell viability compared to Cisplatin alone (Figure 9D). This likely reflects overlapping or independent toxic effects of high-dose vitamin B3 in PC-9 cells (Figure S13 in Supplementary Materials), which complicates the interpretation of potential Cisplatin–B3 interactions in this cell line. The different responses between PC-9 cells and A549 cells highlight that cellular genetic background and metabolic context can influence the outcome of drug–nutrient interactions. To summarize, the obtained in vitro results indicate the time- and concentration-dependent nature of Nicotinamide-related modulation of Cisplatin cytotoxicity and emphasize the complex interplay underlying this interaction.

## 3. Discussion

In the present study, we describe the findings of extensive computational, spectroscopic, and biological studies on the interaction of Cisplatin with Nicotinamide (vitamin B3). Our findings indicate a measurable interaction between Cisplatin and Nicotinamide that is strongly dependent on the chemical environment (i.e., pre-formed complex vs. solution mixture). These results provide mechanistic insight into potential drug–nutrient interactions under controlled in vitro conditions, rather than direct clinical implications. Importantly, the interaction effects described herein were observed predominantly under supra-physiological Nicotinamide concentrations, and therefore their direct relevance to in vivo conditions remains to be established.

The MN15/def2-TZVP level of theoretical calculations shows that the first product of hydrolysis of Cisplatin (CisPt1) has a great affinity for Nicotinamide (B3) via the pyridyl N atom. The build-up of this complex is expected to be thermodynamically favored and exoenergic, with a Gibbs Free Energy (ΔG_r_) equal to −28.70 kcal/mol. This affinity is comparable to that calculated for Cisplatin interactions with DNA nucleobases, e.g., Adenine (−29.34 kcal/mol), indicating that Nicotinamide may act as a potential coordinating ligand under supraphysiological conditions, with the capacity to interact with pharmacologically active Platinum species. These theoretical insights were confirmed experimentally by synthesis and structural characterization of the *cis*-[Pt(NH_3_)_2_NicotinamideCl]NO_3_ complex, which adopts a cis coordination geometry analogous to that found in biologically significant Platinum–ligand complexes. Spectroscopic investigations support the formation of a coordinate covalent bond with the pyridyl N atom showing a broad maximum at 222 nm and a well-defined shoulder at 265 nm. The superior predictive power of MN15, as indicated by MAD values smaller than for B3LYP, also validates the use of MN15 for these types of transition metal–ligand systems.

Also, Platinum(II) can function as a Lewis acid to promote amide bond hydrolysis but this is interesting since such reactivity was reported for some Pt(II) complexes in peptide systems [74,75,76]. Hydrolysis, in these systems, is supposed to be the exception, or that the reaction needs a “well-defined coordination sphere” where the metal center must be at the amide bond, normally through the new position by thiosugar moieties that convey the carbonyl in the right positioning [77,78,79,80]. In the present case, NMR and DFT calculations agree in that Nicotinamide coordinates to Pt(II) via the pyridine nitrogen in a monodentate fashion. In this conformation, the Platinum center is “pulled away” from the amide carbonyl which would reduce the ability to catalyze direct hydrolysis. This is in line with the spectroscopic characterization of the isolated complex, where 1H and 15N NMR clearly confirm the preservation of the amide moiety, without any evidence of transformation to carboxylate.

Meanwhile, it should be pointed out that the Cisplatin aqueous phosphate buffer chemistry is complicated itself [81,82]. Cisplatin is activated by stepwise aquation and ligand exchange mechanisms, taking place within hours under physiological conditions [82,83,84,85]. There is a further equilibrium when phosphate ions are present, since phosphate can transiently bind to Pt(II) to form a dynamic mixture of species. This stabilization induces the conversion of the spectra and allows multiphasic isosbestic points to be disappeared, and the isosbestic points here do not reflect simple two-state transformation in multi-species equilibria.

In this dynamic system, the progressive appearance of spectral features that are consistent with those of the independently prepared *cis*-[Pt(NH_3_)_2_(Nicotinamide)Cl]+ complex, complemented by TD-DFT calculations, suggests the formation of a coordination adduct with Nicotinamide. Nevertheless, as is clear from the complexity of the system, we realize that more than one transient species can be present in solution, and that full speciation would require other complimentary techniques such as LC-MS. Consequently, our conclusion is drawn on the basis of a combination of spectroscopic, computational and synthetic evidence and not through explicit assignment of all species in solution.

One key result of this work is that the biological activity of Cisplatin is severely decreased when it is “caged” in a pre-established complex. In the cell lines A549 and PC-9, the CisPt1-B3 complex showed significantly lower cytotoxic activity (IC_50_ > 125 µM) in comparison to free Cisplatin. Similarly, the complex was found to be nearly completely inactive (MIC ≥ 3600 µM) against a set of bacteria by microbiological plate testing. Our results suggest two potential and non-mutually exclusive mechanisms that may contribute to the observed modulation of Cisplatin activity:

(i) A chemical mechanism, involving coordination of Nicotinamide to reactive Platinum species;
(ii) A biological mechanism, in which Nicotinamide—being a precursor of NAD^+^—may influence cellular pathways related to DNA damage response.

The latter mechanism remains hypothetical and was not directly investigated in the present study. As a whole, these findings suggest a mechanistic pathway for the Cisplatin–Nicotinamide complexation: following intracellular transmembrane transport and hydrolysis-driven conversation to CisPt1, Nicotinamide binds to the electrophilic Platinum center, resulting in a coordination adduct. Discussion of these results in depth by subsections is as follows:

### 3.1. Computational Study

**Figure 10:**
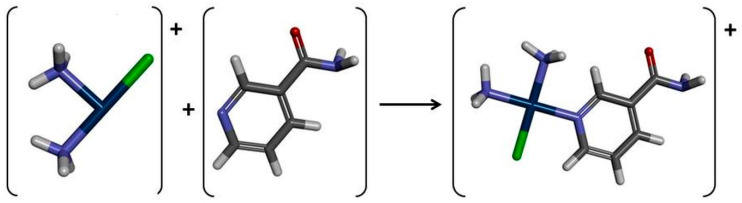
The interaction of mono-aquated forms of Platinum (*cis*-[Pt(NH_3_)_2_Cl]^+^, CisPt1) with Nicotinamide (B3).

Affinity of Cisplatin to Nicotinamide was estimated based on the Gibbs Free Energy of reaction (ΔG_r_) for the complexation’s first product of hydrolysis of Cisplatin (*cis*-[Pt(NH_3_)_2_Cl]^+^, CisPt1) and Nicotinamide (B3) at two levels of calculations, B3LYP/6-31G(d,p)/LANL2DZ and MN15/def2-TZVP (Table 1, Figure 10).

The estimated values were compared to those for the complexation of Cisplatin with the nucleobases in DNA, Adenine and Guanine (Table 1). These thermodynamic values provide crucial insight into the spontaneity and stability of the complexation reactions. Negative values of Gibbs Free Energy of reaction (ΔG_r_) indicate that the complexation reactions are thermodynamically favorable and will proceed spontaneously under the given conditions. At the B3LYP level, the affinity of Cisplatin for Nicotinamide (−26.09 kcal/mol) appears to be greater than for Adenine (−24.72 kcal/mol). However, increasing the level of theory (MN15) changes this interpretation, and both nucleobases, Adenine and Guanine, exhibit a higher affinity for the drug than the tested vitamin (Table 1). At two levels of calculations, B3LYP/6-31G(d,p)/LANL2DZ and MN15/def2-TZV, Guanine has the highest affinity for Cisplatin with Gibbs Free Energy of reaction (ΔG_r_) equal to −27.07 kcal/mol and −31.91 kcal/mol, respectively.

### 3.2. Spectroscopy Results—Theoretical UV-Vis Results and Comparison with Experiment

The theoretical predictions for the Cisplatin-Nicotinamide complex

(*cis*-[Pt(NH_3_)_2_Cl (Nicotinamide)]^+^, CisPt1-B3), alongside their comparison with experimental data, are as follows (Table 2):

- B3LYP/6-31G(d,p)/LANL2DZ

The theoretical λ_max_ predicted by this method for the Cisplatin–Nicotinamide complex is 312.8 nm. The Mean Absolute Deviation (MAD) for this prediction is 23.4, indicating a substantial deviation from the experimentally observed values.

- MN15/def2-TZVP

This method predicts a theoretical λ_max_ of 289.0 nm for the Cisplatin–Nicotinamide complex. The MAD values associated with this prediction are 11.5. These MAD values, whether averaged or taken as a range, are considerably lower than those obtained using B3LYP functional.

Direct comparison for the “average” with the “maximum” MAD clearly indicates that the MN15/def2-TZVP methodology has a better predictive power than the B3LYP/6-31G(d,p)/LANL2DZ for the UV-Vis λ_max_ prediction of the Cisplatin–Nicotinamide complex with an MAD of about 11.5 for MN15 versus 23.4 for B3LYP, the MN15 functional offers the predictions that are on average closer to the experimental values. Such a large step-up in performance implies that MN15, probably because of its more advanced meta-GGA form and the use of a larger and more flexible basis set (def2-TZVP), provides a better representation of the electronic structure, electron correlation, and perhaps key dispersion interactions in this metal–ligand complex. This represents an important observation, as it leads to the strong suggestion that MN15 is the better computational method for future investigations of systems similar to the Platinum–ligand ones considered here.

#### 3.2.1. Analysis of the Experimental UV-Vis Spectrum of *cis*-[Pt(NH_3_)_2_NicotinamideCl]NO_3_

The synthesized complex, *cis*-[Pt(NH_3_)_2_NicotinamideCl]NO_3_, also shows a single and broad absorption band with λ_max_ at ~222 nm in its UV-Vis absorbance spectrum. A well-defined shoulder on the main peak is also observed, protruding into the region at ~265 nm. This spectrum is consistent with the final product of the synthesis.

#### 3.2.2. Analysis and Comparison with the Mixture of Nicotinamide and Cisplatin in the Experiment

Figure 4 depicts the temporal changes in the UV-Vis spectrum of the CisPt–Nicotinamide mixture collected at one time and 168 h. The initial spectrum (at 1 h) contains a peak at about 218 nm and a prominent peak near 266 nm. As the incubation time increases, the peak at 266 nm decreases rapidly and significantly in absorbance; in addition, the intensity and shape of the peak at 218 nm also change. This time-dependent transformation in the UV–Vis spectrum is consistent with a chemical interaction between Nicotinamide and Cisplatin, leading to the formation of new species. The comparison reveals a strong correlation between Figure 4 and Figure 5. The spectral characteristics of the final synthesized complex in Figure 5 (a broad peak with a maximum at around 222 nm and a shoulder at about 265 nm) are very similar to those observed for the mixture in Figure 4. The diminishing absorption in the synthesis (Figure 4) matches the growth of the product, the ultimate spectrum of which is shown in Figure 5. The coexistence of these two discrete absorption regions in Figure 5 clearly indicates that the synthetic reaction generates the *cis*-[Pt(NH_3_)_2_NicotinamideCl]NO_3_. The principal difference between the two figures is that the first one displays the time evolution of the reaction kinetics, whereas the second one depicts the long-time spectrum of the isolated product at the steady state.

#### 3.2.3. Kinetics of Cisplatin–Nicotinamide Complex Formation over Time

**Figure 11:**
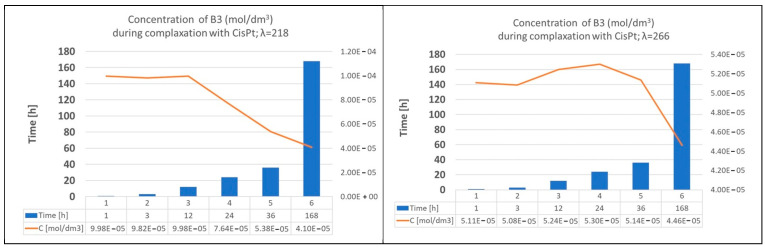
Nicotinamide (B3) concentration during Cisplatin (CisPt) complexation, monitored at wavelengths 218 nm and 266 nm, during incubation at 37 °C in phosphate buffer (pH 7.4).

The kinetics of this complexation are further detailed in Figure 11, which tracks the concentration of Nicotinamide (B3) over time. The concentration of Nicotinamide was calculated based on the standard curves for B3 shown in Figure 5. At the wavelength of 266 nm, the concentration of Nicotinamide initially remains relatively stable, slightly increasing from 5.11 × 10^−5^ mol/dm^3^ at 1 h to a maximum of 5.30 × 10^−5^ mol/dm^3^ at 24 h. After this point, the concentration of Nicotinamide begins to decrease, reaching a value of 4.46 × 10^−5^ mol/dm^3^ at 168 h. This decline in concentration is consistent with the consumption of Nicotinamide as it reacts with Cisplatin to form the complex. A similar trend is observed at λ = 218 nm. The concentration of Nicotinamide remains almost constant for the first 12 h, at approximately 9.9 × 10^−5^ mol/dm^3^. After 12 h, the concentration steadily decreases, dropping to 4.10 × 10^−5^ mol/dm^3^ at 168 h. The decrease in Nicotinamide concentration, particularly after 12–24 h, corresponds to the ongoing formation of the Cisplatin–Nicotinamide complex. The gradual nature of this decrease suggests a time-dependent process for complex formation. While Figure 5 and Figure 11 do not explicitly describe the “breakdown” of the complex, the observed changes in Nicotinamide concentration are consistent with a time-dependent interaction process over time.

#### 3.2.4. Results and Comparison: Cisplatin–Nucleobase Complexes (Cisplatin–Adenine (CisPt1-A) and Cisplatin–Guanine (CisPt1-G))

Experimental UV-Vis Results (Table 2):

- Complex Cisplatin–Adenine (*cis*-[Pt(NH_3_)_2_Cl(Adenine)]^+^, CisPt1-A): Experimental λ_max_ values are reported at 218.0 nm and 265.0 nm.
- Complex Cisplatin–Guanine (*cis*-[Pt(NH_3_)_2_Cl(Guanine)]^+^, CisPt1-G): Experimental λ_max_ values are reported at 220.0 nm and 265.0 nm.

The experimental data show the remarkable similarity in the λ_max_ values for both the Cisplatin–Adenine (218.0 nm, 265.0 nm) and Cisplatin–Guanine (220.0 nm, 265.0 nm) complexes. Despite the distinct chemical structures of Adenine and Guanine (a purine and a keto-purine, respectively), their primary interaction with Cisplatin, as manifested in their UV-Vis spectra, appears to induce very similar electronic transitions or changes in chromophoric properties. This similarity could imply a common dominant binding mode or a similar overall effect on the electronic structure of the nucleobases upon Cisplatin coordination, at least within the resolution and sensitivity of the UV-Vis technique. It suggests that the major chromophoric changes occur in comparable spectral regions for both complexes.

Theoretical UV-Vis Results and Comparison with Experiment (Table 2):

- Complex Cisplatin–Adenine (*cis*-[Pt(NH_3_)_2_Cl(Adenine)]^+^, CisPt1-A):
  ○ B3LYP/6-31G(d,p)/LANL2DZ: Predicted λ_max_ = 316.6 nm, with a MAD of 25.8.
  ○ MN15/def2-TZVP: Predicted λ_max_ = 287.4 nm, with MAD values of 11.2.
- Complex Cisplatin–Guanine (*cis*-[Pt(NH_3_)_2_Cl(Guanine)]^+^, CisPt1-G):
  ○ B3LYP/6-31G(d,p)/LANL2DZ: Predicted λ_max_ = 319.2 nm, with a MAD of 27.1.
  ○ MN15/def2-TZVP: Predicted λ_max_ = 286.8 nm, with MAD values of 10.9.

The computational trend observed for the Cisplatin–Nicotinamide complex is exactly the same for the Cisplatin–Adenine (CisPt1-A) and Cisplatin–Guanine (CisPt1-G) complexes. The MAD values of MN15 (around 10.9 to 11.2) are also significantly lower than those of B3LYP (around 25.8 to 27.1) for the two nucleobase complexes. This strong and consistent behavior observed between a vitamin and two nucleobases with similar types of nitrogenous ligands lends further support to the result that the MN15/def2-TZVP approach is a superior and more dependable computational method to use for the prediction of the UV-Vis spectra of these Pt–ligand complexes. This illustrates the generality and the power of prediction of such a specific theoretical level for these systems.

#### 3.2.5. Cross-Comparison of Nucleobase Complexes

The experimental λ_max_ values for the Cisplatin–Adenine (CisPt1-A) and Cisplatin–Guanine (CisPt1-G) complexes are almost identical, while the theoretical results differ slightly, most notably the MN15 results. For example, MN15 predicts 287.4 nm for the complex of CisPt1-A and 286.8 for the complex of CisPt1-G.

These similarities between the experimental UV-Vis λ_max_ values for the Cisplatin–Adenine and Cisplatin–Guanine complexes (218.0/220.0 nm and 265.0 nm) are so surprising that one could anticipate them to have a very common electronic response to the binding with Cisplatin, but the theoretical predictions, especially by the accurate MN15 functional, expect the λ_max_ values to show subtle differences (for example 287.4 nm for the CisPt1-A and 286.8 nm for the CisPt1-G). While these minute differences might not be distinguished due to the broadness of experimental peaks or inherent experimental errors, such differences illustrate how advanced theoretical methods are capable of capturing subtle variations in electronic structure and excited states due to the distinct molecular design and binding motifs of Adenine versus Guanine with Cisplatin. It also illustrates the balance to be sought between theoretical and experimental approaches: macroscopic evidence from experiments can illuminate the latter at the microscopic level in terms of tiny chemical differences.

#### 3.2.6. Findings and Implications, with Evaluation of Theoretical Method Performance

The most remarkable result from the comparative study is the uniform, dramatic accuracy of MN15/def2-TZVP over B3LYP/6-31G(d,p)/LANL2DZ for the prediction of the UV-Vis λ_max_ values of all the studied Pt–ligands (Cisplatin–Nicotinamide (CisPt1-B3), Cisplatin–Adenine (CisPt1-A), and Cisplatin–Guanine (CisPt1-G)). This can quantitatively be seen through the lower mean MAD values for MN15 (18.6* and 27.1***) than B3LYP (20.4* and 34.6***). Calculating the average MAD values λ_max_ based on the wavelength values λ_max_, only considering those compounds that contain an atom of Pt (Platinum) from the experimental and theoretical works, the highest λ_max_ difference values are strongly observable, i.e., **52.4 and **39.7 in the case of B3LYP and MN15 methods, respectively. The large discrepancy in precision can be explained by the nature of the functionals and basis sets. B3LYP, which is well-established to be a good general functional for many chemical systems, may have some difficulties in accurately describing systems with large charge transfers (like most metal–ligand systems) and in accounting for dispersion forces. The MN15, as a meta-GGA, contains a more elaborate treatment of electron correlation and of non-local effects. These developments are essential for the realistic description of the excited states and the complex interactions in these complexes. In addition, the bigger def2-TZVP basis set is able to give a more flexible and accurate representation of the electron density than the 6-31G(d,p)/LANL2DZ one, especially on the heavy Pt atom, which accounts for the enhanced prediction.

The definitive and repeat performance of MN15/def2-TZVP in providing accurate UV-Vis λ_max_ values for an assortment of Platinum–ligand complexes, which included complexes of ligands with quite different coordination modes, is a very important empirical result and should be giving computational chemists pause. This result gives robust, evidence-based guidance towards the choice of the theoretical method for further studies of related transition metal complexes and their spectroscopic properties.

#### 3.2.7. Interpretation of Spectral Changes and Complex Formation

The experimental UV-Vis spectra clearly indicate the formation of new chemical species upon mixing Cisplatin with Nicotinamide, Adenine, and Guanine. This is evidenced by significant shifts in λ_max_ values and changes in absorbance intensities in Figure 4 and Figure 5 compared to the individual component spectra in Figure S6 in the Supplementary Materials. These changes are characteristic of the formation of new chromophores or alterations of existing ones due to coordination.

The observed spectral changes are a direct consequence of the formation of coordination bonds between Cisplatin and the nitrogenous ligands (Figure 4 and Figure 5). These interactions involve the redistribution of electron density (Figures S6, S9 and S10 in Supplementary Materials, Table S1 in Supplementary Materials). For transition metal complexes, the involvement of d-electron transitions and ligand-to-metal/metal-to-ligand charge transfer bands is often responsible for the observed absorption features.

The experimental protocol involving incubation over 168 h implies that the complex formation is a time-dependent process. The stabilization of spectral features over longer incubation periods would suggest the formation of adducts, whereas continued changes might indicate slower reaction kinetics, the formation of multiple isomers, or degradation pathways.

Beyond simply identifying that complexation occurs, a deeper interpretation of the UV-Vis spectral changes can provide insights into the underlying binding mechanism and strength [86,87]. For example, a pronounced bathochromic (red) shift in the λ_max_ upon complexation often suggests a significant lowering of the energy gap between the highest occupied molecular orbital (HOMO) and the lowest unoccupied molecular orbital (LUMO), which can be indicative of strong charge transfer interactions or extensive electronic delocalization within the newly formed complex (Figures S9 and S10 in Supplementary Materials). Conversely, smaller shifts might suggest weaker interactions or a less dramatic perturbation of the electronic structure. While the provided data primarily lists λ_max_ values, the magnitude of the shift from individual components (if their λ_max_ were explicitly provided in the same table as the complexes) could be correlated with the strength of the metal–ligand bond. Furthermore, the time-dependent nature of the experiment implies that the rate at which these spectral changes occur could be linked to the kinetics of complex formation, providing indirect evidence of how quickly Cisplatin binds to these biomolecules.

### 3.3. Biological Assays

#### 3.3.1. Antimicrobial Activity of Cisplatin

The results show that Cisplatin is active against *E. coli* (MIC 275–450 µM), *P. aeruginosa* (MIC 113 µM), and *K. pneumoniae* (MIC 450–900 µM). This is generally consistent with the literature describing the activity of Cisplatin against Gram-negative bacteria [14,46]. It should be emphasized that the activity against multidrug-resistant strains (ESBL+, VIM-2, OXA-48) is of particular importance and fits well with the current trend to try to repurpose anticancer drugs in addressing antimicrobial resistance [88]. Although the in vitro activity of Cisplatin against these important Gram-negative pathogens, including resistant strains, is encouraging, the MICs (e.g., 113–900 µM) are quite elevated. This represents a major hurdle in clinical translation, as these concentrations could be close to or higher than toxic levels in vivo, and more so given the severe systemic side effects of Cisplatin. The established toxicity of Cisplatin, including nephrotoxicity, neurotoxicity, and myelosuppression [17,18,19,20,21,24,25,26], indicates that the window for therapeutic use of the drug systemically as an antimicrobial agent is likely to be very narrow, if applicable at all, for clinical use. This suggests that it will be difficult to reach efficacious concentrations in vivo without inducing unacceptable adverse effects and leads to the conclusion that systemic repurposing for infections is improbable without either a significant dose reduction or the development of novel delivery strategies.

The MIC values of Cisplatin against *S. aureus* (3600 µM), *E. faecalis* (900–1800 µM), and *B. subtilis* (1800 µM) in the experiment were in general higher than those obtained for Gram-negative bacteria. The literature shows the activity of Cisplatin toward Gram-positive bacteria, such as *S. aureus*, *E. faecalis*, and *Bacillus* species [89]. In particular, the lower MIC values obtained herein for Gram-positive bacteria, in particular for S. aureus (MRSA), which exhibited the highest MIC (3600 µM), deserve attention. The MIC of Cisplatin for *C. albicans* (fluconazole-resistant as well as the ATCC strain) was 1800 µM. The literature clearly reports antifungal activity of Cisplatin towards *C. albicans*, but there is a big quantitative gap. The literature research shows an inhibition at concentrations of 40 μg/mL (about 133 μM) and an activity at 20 μg/mL (approx. 67 μM) for 40 h [90,91,92]. The reported experimental MIC of 1800 µM for Cisplatin against *C. albicans* is considerably higher (13–27 fold) than the concentrations cited in the literature. Such a large discrepancy might be the result of factors like differences in susceptibility of *C. albicans* strains, variation in test methods (broth microdilution versus agar diffusion method), or particular experimental conditions (medium composition, incubation time, inoculum size). This difference needs to be investigated in additional studies to determine and appreciate the actual power of Cisplatin in the case of fungal pathogens and to make sure that results between studies are compatible with each other.

The anti-bacterial activity observed for Cisplatin agrees well with its known mechanism of action, which is primarily acting as a DNA damaging agent (cross linking, intra-strand and inter-strand adduct forming) and via the inhibition of DNA synthesis and cell growth causing cell death. Moreover, Cisplatin also promotes reactive oxygen species (ROS) production, which is also associated with its cytotoxicity. Although these processes are well understood in cancer cells, Cisplatin’s binding to DNA and its effect on the viability of bacterial cells are relatively obscure in the existing literature. This lack of information highlights an aspect that needs to be further explored for a complete understanding of the molecular basis of its antimicrobial activity.

#### 3.3.2. Antimicrobial Activity of Vitamin B3

The observation that there was “no” growth inhibition by vitamin B3 alone at doses of up to 3600 µM (3.6 mM) was at variance with the literature reports [93]. Nicotinamide possesses direct antimicrobial activity and boosts host immunity by promoting the production of antimicrobial peptides (AMPs) [41], as indicated in the literature. For instance, Nicotinamide-induced antimicrobial activity (including morphological alterations in *E. coli* cells) was detected at 2.5% (*w*/*v*) (about 200,000 µM). This is many orders of magnitude above the highest concentration of 3600 µM evaluated in our study.

The difference might be explained by the fact that the concentration of vitamin B3 in our study was too low to detect its individual antimicrobial activity. This methodological constraint limits the quantification of the effect of B3 alone and also restricts the calculation of the FIC index (FICI) for combinations. Therefore, mixture effects have to be interpreted in a qualitative manner and no quantitative FICI values are available from which one could measure the mechanistic effects of mixtures, and thus the depth of these effects cannot be qualitatively determined. Although the direct activity was not observed in this report, Nicotinamide’s antimicrobial mechanisms are well-described in the literature. It induces microbial cell cycle arrest without leading to productive cellular division, resulting in enlarged cells. Nicotinamide also disorganizes and disperses bacterial DNA and directly binds to and breaks DNA, impairing replication. It is a PARP-1 inhibitor, i.e., an enzyme that can modulate the DNA repair process. In addition, as an NAD precursor, Nicotinamide could influence bacterial metabolism and also, at certain concentrations, the bacterial susceptibility to antibiotics.

#### 3.3.3. Interactions of Cisplatin and Vitamin B3

The qualitative assessment (Table 3, Table 4 and Table 5) comparing the MIC of the “CisPt+B3” mixture (further: mixture) with the MIC of Cisplatin alone reveals varied interactions.

- Synergism: Observed for *P. aeruginosa* ATCC 27853 (CisPt MIC 113 µM vs. mixture 57 µM), *K. pneumoniae* ATCC 700603 (CisPt MIC 900 µM vs. mixture 450 µM), and for both *C. albicans* strains (CisPt MIC 1800 µM vs. mixture 900 µM). This indicates that for these strains, the mixture is more effective than Cisplatin monotherapy.
- Antagonism: Observed for *E. coli* strains (e.g., ECO ATCC 25922: CisPt 450 µM vs. mixture 1800 µM), *E. faecalis* strains (e.g., EFA ATCC 29212: CisPt 900 µM vs. mixture 1800 µM), *P. aeruginosa* 1 (VIM-2) (CisPt 113 µM vs. mixture 225 µM), and *K. pneumoniae* 1 (OXA-48) (CisPt 450 µM vs. mixture 1800 µM).
- Indifference/Additivity: Observed for *S. aureus* strains (3600 µM for both CisPt and mixture) and *B. subtilis* strains (1800 µM for both CisPt and mixture).

The stark contrast in the activities of the synthesized complex (usually very low activity, MIC ≥ 3600 µM) and the “CisPt+B3” freshly prepared mixture (showing well-defined but variable MIC values) is an important observation. This suggests that pre-formed complexes could render Cisplatin less active or less bioavailable under the conditions of the assay. On the other hand, a newly made solution, with CisPt and B3 separately added, enables them to act on their own or to have dynamic, transient interactions that are more potent. The presence of several multidrug-resistant strains (including ESBL+, MRSA, VRE, VIM_2, OXA_48, and fluconazole-resistant *C. albicans*) in the investigation is highly relevant in terms of the antimicrobial resistance crisis [94]. The idea of “collateral sensitivity” in which resistance to one drug creates increased sensitivity to another is also becoming more popular to combat resistance. The outcomes are conflicting (synergism and antagonism) for particular resistant strains (e.g., synergy for PAE ATCC, antagonism for PAE 1 (VIM-2); synergy for KPN ATCC and antagonism for KPN 1 (OXA-48)) strongly indicate that the effectiveness of the combination would be closely related to the particular resistance mechanism present within a strain, and this means that a generic “CisPt+B3” combination might not work for all forms of resistance. It indicates that a more refined understanding of resistance mechanisms and possibly personalized antimicrobial approaches, where the underlying molecular resistance pathways could be exploited for synergism or contribute to detrimental antagonism, may be necessary.

There is strong support in the literature for the concept of combination therapy to counteract resistance and increase efficacy, especially in anticancer chemotherapy [95]. Nicotinamide was indicated to sensitize human breast cancer cells to the cytotoxicity of Cisplatin acting as a PARP inhibitor [96]. Although this is in a tumoral context, it just shows a biological interaction of CisPt and Nicotinamide with its precellular effects.

### 3.4. Cell Line Study

The effect of vitamin B3 on the antitumor activity of Cisplatin (CisPt) was evaluated in A549 and PC-9 cell lines using three experimental models. In the first model (Figure 6 and Figure 7), vitamin B3 was administered every 24 h for a total duration of 48 h (A), 24 h (B), or 48 h (C), with a final dose applied concurrently with Cisplatin. In the second model (Figure 8), vitamin B3 and CisPt were administered simultaneously for 24 h (A,B) or 48 h (C,D). In the third model (Figure 9), cells were pre-treated with vitamin B3 for 24 h followed by Cisplatin exposure for either 24 h (A,B) or 48 h (C,D).

It should be stressed that the performed investigations were preclinical in nature, conducted in in vitro models using NSCLC cell lines, and are therefore not directly translatable to clinical settings. The inclusion of elevated doses of vitamin B3 (2–8 mM) in these experiments was necessary to evaluate potential coordination tendencies and underlying interaction mechanisms in vitro. These values differ substantially from the concentrations associated with fasting plasma Nicotinamide levels of ~5 μM [97,98,99] or even those reached in vivo under normal conditions, which are ~69 μM [98,100].

Cell line detail observations point to an intricate interplay between Cisplatin and Nicotinamide that may have critical ramifications in cancer treatment. The CisPt1-B3 complex, however, displayed an almost uniformly lower cytotoxicity than Cisplatin in both NSCLC cell lines (A549, PC-9). In the case of A549 cells, the IC_50_ values of the CisPt1-B3 complex were always over 125 µM for all incubation times tested, which shows a great reduction in potency as compared to the free drug Cisplatin, whose IC_50_ values were within the low micromolar range. The complex, although it did become active at later time points, remained far less active overall, and in PC-9 cells as well. These results indicate that complexation may decrease the bioavailability and/or the efficacy of Cisplatin in the cell and that this particular methodology for complexation may not be suitable for increasing the anticancer activity of Cisplatin.

Pre-incubation or co-treatment with Nicotinamide and Cisplatin produced cell line-specific effects. In A549 cells, high concentrations of Nicotinamide (2–4 mM) administered 24–48 h prior to Cisplatin partially reduced Cisplatin cytotoxicity, consistent with a threshold-dependent rather than linear dose–response relationship. This effect may reflect Nicotinamide’s involvement in DNA damage response pathways (e.g., as an NAD^+^ precursor or via PARP-1 modulation), potentially enhancing repair of Cisplatin-induced DNA lesions. However, no direct measurements of DNA damage or repair (e.g., γH2AX or PARylation assays) were performed, and therefore this interpretation remains speculative. These findings underscore a possible drug–nutrient interaction relevant for patients receiving Cisplatin-based chemotherapy while supplementing with B vitamins, warranting further investigation in relevant models, in the near future. In PC-9 cells, responses were more variable. Certain treatment regimens (e.g., B3 pre-treatment followed by 48 h co-incubation with Cisplatin) showed mild antagonism, whereas shorter pre-treatment or 24 h co-treatment did not reduce Cisplatin cytotoxicity. At higher Nicotinamide concentrations (4–8 mM), apparent reductions in cell viability were observed, likely reflecting the inherent cytotoxicity of B3 rather than specific pharmacological interactions. Overall, these results suggest that the effects of Nicotinamide on Cisplatin activity are dependent on concentration, treatment sequence, and exposure time.

Overall, among the different treatment regimens, the most pronounced changes in Cisplatin cytotoxicity were observed when cells underwent prolonged Nicotinamide pre-treatment followed by co-treatment with B3 and Cisplatin for 48 h. These conditions led to a noticeable reduction in Cisplatin’s anticancer activity, highlighting the impact of both exposure duration and treatment sequence.

This study was designed to evaluate global alterations in the cytotoxic capacity of Cisplatin in the presence of vitamin B3, and not to study specific molecular pathways of DNA repair. The high concentration of vitamin B3 (Nicotinamide) used in these experiments was warranted to visualize the molecular coordination tendencies. Overall, the results of the study suggest that under specific conditions, co-incubation with vitamin B3 may partially reduce the in vitro efficacy of the chemotherapeutic drug. This biological modulation may be due to the fact that Nicotinamide is a precursor of NAD^+^, and thus may affect the PARP-1-dependent DNA repair process. Although study is focused on cytotoxicity, it is still possible that the NAD^+^/PARP-1-mediated axis is involved, being a hypothesis that remains to be assessed in further studies to elucidate molecular interactions. These results indicate a potential danger of drug–nutrient interactions which should be confirmed in further in vivo studies to assess the clinical relevance.

Eventually, the distinct and multifaceted responses in PC-9 versus A549 cells highlight the necessity to account for genetic background and metabolism in the assessment of drug–drug interactions. Mutations in KRAS, KEAP1, KMT2C, and FAT4 are present in A549 while in PC-9 mutations in TP53, CDKN2A, and EGFR are found. PC-9 cells were more susceptible to Cisplatin than A549 cells. This underlines an increasing importance of personalized medicine in oncology and the forte that drug–nutrient interactions may be cell line- or patient-specific, and therefore tailor-made solutions will have to be developed. In the end, these data indicate that, even if Cisplatin is a strong anticancer drug, its activity could be influenced by Nicotinamide, and moreover the influence could be altered by dosage, administration schedule, and genetic background. These findings highlight the need for further investigation of potential drug–nutrient interactions in more clinically relevant settings, in the near future.

## 4. Methods and Materials

### 4.1. Chemistry (Synthesis Part)

#### 4.1.1. Materials

All analytical grade chemicals and reagents were purchased from Merck (Poland, Warsaw, Poland). Chemicals were used without further purification, and their purity was as required for spectroscopic studies (≥99%).

#### 4.1.2. Synthesis of the *cis*-[Pt(NH_3_)_2_NicotinamideCl]NO_3_ Complex

General method for the synthesis of the *cis*-[Pt(NH_3_)_2_NicotinamideCl]NO_3_ complex

**Scheme 1:**
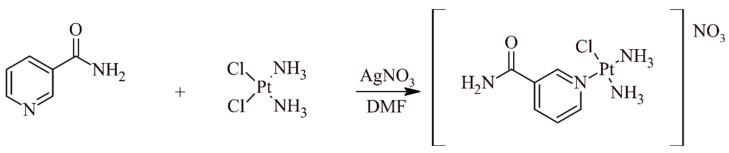
The schematic representation of synthesis of the *cis*-[Pt(NH_3_)_2_NicotinamideCl]NO_3_ complex.

The nitrate salt of the monofunctional Cisplatin complex with Nicotinamide was prepared through a modification of a previously reported method [45]. A solution of AgNO_3_ (0.68 g, 4 mmol) was added dropwise to a solution of Cisplatin (1.2 g, 4 mmol) in 20 mL DMF. The reaction mixture was stirred at 60 °C in the dark for 4 h, after which the AgCl was filtered. Subsequently, Nicotinamide (0.44 g, 0.36 mmol) was added to the reaction mixture, which was then stirred at 60 °C overnight. The reaction mixture was then evaporated under reduced pressure, and the residue was dissolved in 30 mL MeOH. The unreacted Cisplatin was removed through filtration. The resulting supernatant was vigorously stirred, followed by the addition of 100 mL of diethyl ether to induce precipitation of the desired solid compound. The precipitate was collected by filtration and washed twice with 20 mL of diethyl ether. The compound was purified by dissolving in methanol and precipitating it dropwise into vigorously stirred diethyl ether. Finally, the compound was isolated via vacuum filtration and dried under reduced pressure. The general route for the preparation of the *cis*-[Pt(NH_3_)_2_NicotinamideCl]NO_3_ complex is shown in Scheme 1.

#### 4.1.3. Characteristic of Synthesized Complex

The synthesized compound was characterized by comprehensive 1D and 2D NMR spectroscopy (^1^H, ^13^C, ^15^N, HSQC, and HMBC). Representative spectra are included in the Supplementary Material (Figures S2 and S3).

**Scheme 2:**
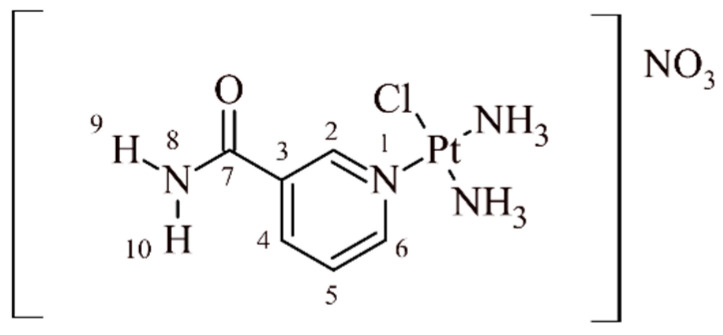
Structure and atom numbering in *cis*-[Pt(NH_3_)_2_NicotinamideCl]NO_3_ complex.

*cis*-[Pt(NH_3_)_2_NicotinamideCl]NO_3_ (Scheme 2), white solid, yield: 51%. ^1^H NMR (DMSO-*d*_6_ from TMS) *δ*: 9.13 (s, 1H, H-2), 8.84 (d, ^3^*J*_H,H_ = 5.36 Hz, 1H, H-6), 8.42 (d, ^3^*J*_H,H_ = 8.04 Hz, 1H, H-4), 8.37 (s, 1H, H-9), 7.90 (s, 1H, H-10), 7.68 (t, 1H, H-5), 4.65 (s, 3H, NH_3_), 4.33 (s, 3H, NH_3_). ^13^C NMR (DMSO-*d*_6_ from TMS) *δ*: 164.7 (C-7), 155.4 (C-6), 152.9 (C-2), 137.9 (C-4), 132.5 (C-3), 126.3 (C-5). ^15^N NMR (DMSO-*d*_6_ from MeNO_2_) *δ*: −422.9 (NH_3_), −414.8 (NH_3_), −270.5 (N-8), −169.8 (N-1). IR (ATR) (cm^−1^): 3379–3188 m (ν_N–H_ and ν_C–H(Ar)_), 1668 s (ν_C=O_).

#### 4.1.4. NMR Spectra

The ^1^H and ^13^C NMR spectra were measured on a Bruker Ascend III (Bydgoszcz, Poland) spectrometer operating at 400 MHz. Dimethyl sulfoxide served as the solvent, while tetramethylsilane (TMS) for ^1^H and ^13^C and nitromethane (CD_3_NO_2_) for ^15^N were used as internal standards. Chemical shifts (*δ*) are reported in ppm relative to TMS/CD_3_NO_2_ and coupling constants (*J*) are expressed in Hz (Figures S2 and S3 in Supplementary Materials).

#### 4.1.5. Differential Scanning Calorimetry (DSC) of the *cis*-[Pt(NH_3_)_2_NicotinamideCl]NO_3_ Complex

To investigate the properties of the synthesized *cis*-[Pt(NH_3_)_2_NicotinamideCl]NO_3_ complex (Cisplatin–Nicotinamide, CisPt1-B3), differential scanning calorimetry (DSC) analysis was performed. The melting temperature of the synthesized complex was 192.79 °C (Figure S4 in the Supplementary Materials).

### 4.2. Computational Methods

The computational approach was based on density functional theory (DFT) [101,102] to evaluate and interpret the geometric, energetic, and electronic characteristics of the studied compounds. Molecular structures were initially constructed and visualized using GaussView 6.0.16 software [103], which facilitated the setup of the models. Quantum-chemical calculations were performed with Gaussian 16 Rev. C.01 [103]. To optimize molecular geometries and locate energy minima, two different levels of theory were employed B3LYP/6-31G(d,p) and MN15/def2-TZVP:

- B3LYP [104,105]: This hybrid functional combines Becke’s 1988 exchange functional [106] with the Lee–Yang–Parr correlation functional [107], incorporating a portion of Hartree–Fock exchange. It is commonly used due to its versatility across various molecular systems. For heavy atoms such as Platinum, the LanL2DZ basis set was applied [108], which includes relativistic effective core potentials to account for the effects of heavy elements.
- MN15 [102,109]: This functional is tailored for complex systems involving significant electronic interactions and larger molecular structures. It employs a different exchange-correlation scheme providing enhanced accuracy and broader applicability, especially for multi-electron systems.

This multi-level computational methodology was selected to ensure robust and comparable results, aligning with previous studies on compounds such as Cisplatin and nucleobases, where B3LYP-based calculations yielded results consistent with experimental data [110]. However, the selection of a DFT functional and basis set is critically important for the accuracy of computational predictions. Different functionals incorporate varying approximations for the exchange-correlation energy, which represents the most challenging component of DFT calculations. Consequently, their performance can differ significantly depending on the specific chemical system under investigation (e.g., metal complexes versus purely organic molecules) and the particular property being calculated (e.g., ground state geometries versus excited state energies for UV-Vis spectra). By employing and comparing two distinct computational levels, B3LYP and MN15, the reliability and robustness of their theoretical predictions can be rigorously assessed. This comparative analysis helps to identify which functional provides a more accurate and physically sound description of the electronic structure and excited states pertinent to these Platinum–ligand interactions. The MN15 functional was specifically employed due to its superior performance in treating noncovalent interactions and its parameterization for transition metals, which is critical for an accurate description of the Platinum(II) coordination sphere compared to the standard B3LYP functional. Using multiple levels of theory enables a more comprehensive analysis and validation of the theoretical models [50,51,52,54], for which the Mean Absolute Deviation (MAD) values serve as a quantitative metric, directly guiding the choice of methods for future, more extensive computational investigations, for which the Mean Absolute Deviation (MAD) values serve as a quantitative metric, directly guiding the choice of methods for future, more extensive computational investigations [111].

Energy minimizations at both levels were conducted to identify stable structural conformations. Harmonic vibrational frequency calculations were performed to determine Zero Point Energies (ZPEs) and to verify that the optimized structures represent true minima on the potential energy surface, as indicated by the absence of imaginary frequencies. It is important to note that only a single tautomeric and conformational form of each Drug–Nicotinamide (nucleobases) complex was considered in this study. While this ensures consistency in comparing binding affinities, the potential impact of alternative tautomers or conformers remains an acknowledged limitation and will be explored in future research.

Solvent effects of water were incorporated using the Polarizable Continuum Model (IEF-PCM), with radii based on the Bondi scheme [112], to simulate an aqueous environment and account for solvation. This implicit solvation model treats the solvent as a polarizable dielectric continuum, providing an efficient approximation of bulk water effects suitable for relative affinity assessments. Although it does not explicitly model specific solute–solvent interactions like hydrogen bonding, it offers a practical balance between accuracy and computational efficiency for the comparative analyses presented [113,114,115,116].

Spectroscopic properties were calculated using the PBE0 hybrid functional, recognized for its reliable prediction of electronic excitation spectra [117].

The HOMO and LUMO energies [118], which are fundamental for understanding electron distribution and potential electronic transitions, were calculated using the same DFT methods (B3LYP/6-31G(d,p)/LANL2DZ and MN15/def2-TZVP). These frontier orbital energies are critical as they directly relate to a molecule’s ability to donate (HOMO) or accept (LUMO) electrons, thereby influencing its chemical reactivity.

From these calculated HOMO and LUMO energies, a suite of chemical reactivity descriptors was derived. These parameters provide quantitative insights into a molecule’s chemical behavior:

- Energy Gap (ΔE_gap_): The energy difference between the LUMO and HOMO (LUMO–HOMO), which serves as an indicator of molecular stability and reactivity. A smaller gap typically implies higher reactivity and easier electronic transitions.
- Absolute Electronegativity (χ): Represents the power of an atom or group of atoms to attract electrons. It is calculated as the negative of the chemical potential.
- Chemical Potential (μ): Indicates the escaping tendency of electrons from a system.
- Absolute Hardness (η): A measure of resistance to charge transfer or deformation of the electron cloud. A larger value indicates a harder (less reactive) molecule.
- Absolute Softness (σ): The inverse of hardness, indicating the ease of charge transfer.
- Global Electrophilicity (ω): Quantifies the tendency of a molecule to accept electrons. A higher value indicates a stronger electrophile.
- Global Softness (S): Another measure related to the inverse of hardness.
- Additional Electronic Charge (ΔN_max_): Represents the maximum amount of electronic charge that a molecule can accept.

These derived parameters offer a comprehensive view of how the electronic properties of Cisplatin and its ligands change upon complexation, providing a microscopic basis for understanding their altered chemical behavior and spectroscopic characteristics.

### 4.3. Spectroscopy

The experimental UV-Vis spectroscopic studies were conducted under carefully controlled conditions designed to mimic physiological environments:

- Incubation Conditions: All experimental mixtures, including those of Nucleobase (Adenine, Guanine)–Cisplatin and Cisplatin–Nicotinamide, were incubated at 37 °C in a phosphate buffer maintained at a pH of 7.4. These conditions were chosen to simulate the in vivo environment, thereby ensuring the biological relevance of the observed interactions and spectral changes.
- Time-Dependent Measurements: To monitor the progression of complex formation and assess the stability of the formed complexes, samples were collected at specific time points: 1, 3, 12, 24, 36, and 168 h.
- Control Samples: For comparative purposes, control samples containing only Cisplatin, Nicotinamide, Adenine, or Guanine were prepared. These controls were dissolved in phosphate buffer (pH 7.4) at identical concentrations to the experimental mixtures. The inclusion of these controls is crucial for differentiating specific spectral changes that arise from Cisplatin–ligand complexation from potential background absorbance or non-specific interactions.

The concentration of Cisplatin used in the UV-Vis spectroscopy study (3.23 µM), as well as the range applied in cellular experiments (0.04 µM to 125 µM), was selected to reflect clinically relevant plasma levels. The applied Cisplatin concentrations are in line with previously reported in vitro studies using similar cancer cell lines, where effective concentrations ranged from low micromolar to over 100 µM, depending on exposure time and cell sensitivity [119,120]. According to pharmacokinetic data, standard therapeutic doses (25–100 mg/m^2^) result in comparable concentrations [9,121,122,123,124]. However, the intracellular accumulation of Cisplatin is influenced not only by the administered dose but also by factors such as exposure time and the level of cellular resistance to the drug [125].

The assumptions were verified against data reported in the literature, where a therapeutic dose of 100 mg (63 mg/m^2^) of Cisplatin was administered, and pharmacokinetic analyses were performed based on Platinum concentrations. Following this dosage, peak plasma levels of filterable Platinum reached 1120 ng/mL (5.7 µM), while total Platinum levels reached 1280 ng/mL (6.6 µM) [126]. In further studies, IC_50_ values of Cisplatin were determined for several human non-small cell lung cancer cell lines: A549 (1.58 µM), SKMES-1 (4.09 µM), MOR (6.39 µM), and H460 (5.72 µM) [127]. Additionally, total Platinum accumulation in colon cancer cell lines was found to range from 27 to 59 µM [128]. These findings are consistent with the initial assumptions.

In the study, Nicotinamide was applied at a concentration of 6.53 µM, corresponding to a 2:1 molar ratio relative to Cisplatin. This concentration is consistent with typical fasting plasma Nicotinamide levels, which are approximately 5 µM [97]. Under normal physiological conditions, Nicotinamide concentrations in human blood generally reach around 69 µM [100]. Therefore, the concentrations used in the UV-Vis experiments remain within the physiological range.

### 4.4. Biological Assays

The microbiological investigations included a broad spectrum of clinically relevant strains, encompassing ATCC reference strains and antibiotic-resistant strains. This comprehensive strain selection is crucial for assessing the therapeutic potential of the tested substances. The microorganisms studied were as follows:

- Gram-negative bacteria: *Escherichia coli* (ECO ATCC 25922 and ECO1 (ESBL+ strain)), *Pseudomonas aeruginosa* (PAE ATCC 27853 and PAE 1 (VIM-2 strain)), *Klebsiella pneumoniae* (KPN ATCC 700603 and KPN 1 (OXA-48 strain)).
- Gram-positive bacteria: *Staphylococcus aureus* (SAU ATCC 29213 and SAU1 (methicillin resistant strain, MRSA)), *Enterococcus faecalis* (EFA ATCC 29212 and EFA 1 (vancomycin resistant strain), VRE), *Bacillus subtilis* (BSU ATCC 7972 and BSU 1).
- Fungi: *Candida albicans* (CAL ATCC 90028 and CAL 1 (fluconazole-resistant strain)).

The studies utilized three main forms of substances: Cisplatin (CisPt), vitamin B3 (B3), and their mixtures:

- CisPt (Cisplatin): 108.4 mg of CisPt was dissolved in 100 mL of Mueller–Hinton Broth (MHB), yielding a concentration of 3.6 mM.
- B3 (vitamin B3): In the first stage of anti-microbiological testing, 44.0 mg of B3 was prepared and dissolved in 100 mL MHB, corresponding to a concentration of 3.6 mM. In the second stage, the weighing of B3 was 88.0 mg in 100 mL MHB, which, combined with 108.4 mg CisPt, gave a molar ratio of 1:2 (3.6 mM CisPt: 7.2 mM B3).
- The synthesized CisPt1-B3 complex: In the first stage of anti-microbiological testing, a synthesized complex, prepared from 161.6 mg, dissolved in 100 mL MHB, was used.
- CisPt+B3 (fresh mixture): In the second stage of anti-microbiological testing, CisPt (108.4 mg) and B3 (88.0 mg) were placed in a single tube and poured with 100 mL of sterile MHB, and then dissolved together. The molar ratio in this mixture was 1:2 (3.6 mM CisPt: 7.2 mM B3).

The minimum inhibitory concentration (MIC) was defined as the lowest concentration of the tested substance at which no macroscopic growth of microorganisms was observed (the medium remained clear). The MIC determination procedure was performed according to Clinical and Laboratory Standards Institute (CLSI) standards. A series of two-fold dilutions of the tested substances in Mueller–Hinton Broth (MHB) (Becton Dickinson, Franklin Lakes, NJ, USA) were prepared. The concentration range for CisPt, B3, and the CisPt1-B3 complex was from 3600 µM down to 1.75 µM or 2.15 µM. For the CisPt+B3 mixture, dilutions ranged from 3600 µM: 7200 µM (CisPt: B3) down to 2.15 µM: 4.30 µM (CisPt: B3). Microbial suspensions were prepared to an optical density of 0.5 McFarland scale in sterile 0.9% NaCl. To the wells of a multi-well plate, 200 µL of the appropriate concentration of the tested substance in MHB and 2 µL of the strain suspension in 0.9% NaCl were added. Positive controls (200 µL sterile MHB + 2 µL strain suspension) were included to confirm bacteria growth without the tested substance, and negative controls (200 µL of the given concentration of the tested substance in MHB + 2 µL sterile NaCl) were prepared. Plates were incubated for 20–24 h at 35 °C in a humid chamber. The entire experiment was performed in three replicates, ensuring the reliability and reproducibility of results.

A key methodological difference between the first and the second stages of anti-microbiological testing is the preparation method of the combined substances. In the first stage, the “Complex” (161.6 mg) was prepared as a separate weighed amount and dissolved separately in 100 mL of MHB, suggesting that it is a pre-synthesized CisPt1-B3 complex. In contrast, in the second stage, CisPt (108.4 mg) and B3(88.0 mg) were weighed into a single tube, and then poured with 100 mL of sterile MHB and dissolved together, forming a freshly prepared mixture in a 1:2 molar ratio. This fundamental difference in the preparation method and the resulting chemical state is extremely important for interpreting the observed antimicrobial activities. The stability, solubility, chemical reactivity, and, consequently, the bioavailability and biological activity of a pre-formed complex can significantly differ from a simple solution containing the same components. This distinction is crucial for understanding the observed variations in antimicrobial efficacy, highlighting that the chemical state and immediate interactions between Cisplatin and Nicotinamide profoundly influence their biological activity.

### 4.5. Cell Studies (Lung Cancer Cell Line Studies)

In vitro studies were conducted on two human non-small cell lung cancer (NSCLC) cell lines: A549 and PC-9. A549 cells exhibit KRAS, KEAP1, KMT2C, and FAT4 mutations, while PC-9 cells harbor mutations in TP53, CDKN2A, and EGFR. A549 and PC-9 human non-small cell lung cancer cell lines were cultured in RPMI-1640 medium (Biowest, Nuaillé, France, cat. no. L0501-500) supplemented with 10% fetal bovine serum (FBS; Biowest, cat. no. S181BH-500), 2 mM L-glutamine (Biowest, Nuaillé, France, cat. no. X0550-100), and 1% penicillin–streptomycin solution (100×; Biowest, Nuaillé, France, cat. no. L0022-100). Cells were maintained at 37 °C in a humidified incubator with 5% CO_2_ and passaged upon reaching 80–90% confluency. The cytotoxic effects of Nicotinamide (vitamin B3), Cisplatin (CisPt, CDDP), the pre-formed CisPt1-B3complex, and combinations involving Nicotinamide pre-incubation or co-treatment with Cisplatin (CisPt, CDDP) were evaluated using the MTT assay over 24, 48, and 72 h. For this purpose, a series of in vitro experiments was conducted, involving different treatment schemes and response analyses.

#### 4.5.1. Preparation of Compound Solutions

Solutions of Nicotinamide (80 mM), Cisplatin (3 mM), and their complexes (3 mM) were freshly prepared in ultrapure water (Millipore), using ultrasonication for uniform dissolution, and sterilized by filtration through 0.22 µm syringe filters. Tested concentrations ranged from 0.04 µM to 125 µM, with Nicotinamide solutions reaching up to 8 mM at higher concentrations. During the preparation of the final mixtures, solutions were kept on ice and protected from light.

#### 4.5.2. Treatment Schemes

Lung cancer cells were seeded in 96-well plates at a density of 5000 cells/cm^2^. The following day, they were subjected to the treatment protocols as described in detail below. Control cells were incubated in culture medium supplemented with sterile water at the same volume ratio used for the experimental treatment groups. The experiments included various temporal and sequential treatment protocols:

- Direct effect of different compound concentrations on cell growth: To assess cytotoxicity, cells were treated with 100 µL of Cisplatin (CisPt), the synthesized complex (CisPt-B3 complex), or a freshly prepared mixture (CisPt+B3 Mix). For Cisplatin and the synthesized complex, final concentrations were 0, 0.04, 0.2, 1, 5, 25, and 125 µM. In the groups involving the combination of both compounds (CisPt+B3 Mix), a constant 1:2 molar ratio (Cisplatin to Nicotinamide) was maintained. Consequently, in the mixture, vitamin B3 concentrations were 0, 0.08, 0.4, 2, 10, 50, and 250 µM, respectively. Cells were incubated for 24, 48, or 72 h before viability assessment.
- Sequential pre-treatment with Nicotinamide followed by Cisplatin: Cells were incubated with Nicotinamide (B3) at final concentrations of 0, 0.125, 0.5, 1, 1.5, or 2 mM, with B3 supplemented every 24 h for a total of 24 or 48 h. After this pre-treatment period, Cisplatin (CisPt) was added alone or with the concurrent addition of B3 to assess combined exposure effects. After 24 or 48 h, the cell viability was assessed.
- Concurrent (parallel) treatment with Nicotinamide and Cisplatin: Cells were treated with B3 (0, 0.25, 0.5, 1, 2, 4 mM) and Cisplatin simultaneously for either 24 h or 48 h (without prior B3 pre-incubation).
- Pre-treatment with Nicotinamide followed by Cisplatin-only treatment: Cells were incubated with B3 (0, 0.25, 0.5, 1, 2, 4, 8 mM) for 24 h, after which Cisplatin was added for either 24 h or 48 h.

#### 4.5.3. Assessment of Cytotoxicity and Data Analysis

The cytotoxic effects were evaluated using the MTT assay, according to standard protocols. A 5 mg/mL MTT solution was prepared in PBS, sterilized, and stored at −20 °C. At the end of the experiment, the MTT solution was added to the culture medium to a final concentration of 0.5 mg/mL. Cells were incubated for 3.5 h in a CO_2_ incubator. After incubation with MTT, the medium was removed, and isopropanol with HCl was added to dissolve formazan crystals. Plates were incubated for 10 min with shaking at 250 rpm at room temperature. Absorbance was measured spectrophotometrically at 570 nm, with a correction at 690 nm, using a Synergy H1 Multiskan Spectrum BioTek (Winooski, VT, USA) microplate reader. Results were expressed as a percentage of cell viability relative to controls. Statistical significance was assessed using one-way ANOVA followed by Dunnett’s post hoc test or two-way ANOVA followed by Tukey’s post hoc test, with each treatment group compared to the Cisplatin-only control. The data are expressed as means ± standard error of the mean (SEM). A *p*-value of less than 0.05 was considered statistically significant. Significance levels are indicated on the figures as follows: * *p* < 0.05, ** *p* < 0.01, *** *p* < 0.001, and **** *p* < 0.0001. IC_50_ values were calculated via nonlinear regression analysis using GraphPad Prism 9.2.

## 5. Study Limitations

Although this work provides a multiscale assessment of Cisplatin (CisPt) interactions with Nicotinamide (B3), several limitations should be acknowledged. Firstly, in the part of thermodynamic and structural studies, all of our spectroscopic and computational studies were performed on the thermodynamic and structural aspects of the *cis*-[Pt(NH_3_)_2_NicotinamideCl]NO_3_ complex and its formation in phosphate buffer. The UV–Vis results and NMR spectra support the formation of new chemical species after the coordination via pyridine nitrogen; however, detailed speciation of interaction products in complex biological environments (e.g., cell culture media containing competing nucleophiles) was not performed. In addition, the Nicotinamide concentrations employed in our in vitro biological experiments (250 µM to 8 mM) are several orders of magnitude above those found in normal human serum, which typically does not exceed ~65 µM even with supplementation. These supra-physiological levels were selected to enable detection of interaction phenomena under controlled in vitro conditions that may not be observable at lower concentrations. Thus, it is important to note that although our findings suggest useful mechanistic insights into drug–nutrient relations involving reactive Platinum species, the effects observed in cell culture may not directly translate to clinical conditions, and require further research. Additional in vivo studies with clinically relevant dosing schedules are required to establish the true effect of vitamin B3 supplementation on the treatment outcomes of Platinum-based chemotherapy.

Additionally, kinetic aspects of complex formation under physiologically relevant timescales were not directly evaluated, and the influence of competing biomolecules (e.g., proteins, thiols, and chloride ions) present in vivo was not addressed. Future studies should aim to integrate these factors to better define the relevance of the observed interactions under biologically and clinically relevant conditions.

Furthermore, no direct measurements of intracellular Cisplatin–Nicotinamide complexes were performed (e.g., LC-MS or in situ spectroscopic techniques), and no direct assessment of DNA damage or repair processes (such as γH2AX foci formation or PARP activity assay) was conducted. Therefore, the proposed biological mechanisms remain hypothetical.

Overall the results presented above are consistent with a chemically reasonable interaction between Cisplatin and Nicotinamide under the conditions used, but the statement should be made that the complexity of solution species in biological environments is yet to be clarified.

## 6. Conclusions

The results of this multi-tiered study provide a combined chemical and biological assessment of the interaction between Cisplatin and Nicotinamide (vitamin B3). Using advanced theoretical chemical methods combined with in vitro activity assays, our findings support the hypothesis that Nicotinamide undergoes coordination processes with Platinum species. These findings support the existence of a potential drug–nutrient interaction under controlled in vitro conditions, which may influence the activity of Platinum-based chemotherapeutics.

A key synthesis of our results suggests the hypothesis that the modification of drug bioactivity occurs through underlying molecular interaction mechanisms. High-level MN15/def2-TZVP calculations confirmed the exergonic nature of the complexation (ΔG_r_ = −28.70 kcal/mol), showing an affinity comparable to that of Cisplatin for Adenine. This suggests that Nicotinamide can interact with reactive Platinum species, a phenomenon supported by the isolation and structural elucidation of the *cis*-Pt(NH_3_)_2_NicotinamideCl]NO_3_ complex. Biologically, this effect is associated with a substantial reduction in cytotoxicity (IC50 > 125 μM) relative to unbound Cisplatin. Lowered bioactivity of the pre-formed complex is in line with a Pt–Nicotinamide coordination bond formation at aqueous buffered condition (pH 7.4) that could reduce the concentration of pharmacologically active Platinum species.

In addition, our data reveal an essential difference between “pre-formed complexes” and “freshly mixed solutions.” While the synthesized complex is inactive, antagonism to synergism is observed in the dynamic interactions in freshly prepared solutions. These observations suggest that the timing and conditions of exposure may influence the interaction between Nicotinamide and Cisplatin under experimental conditions. Apart from straightforward chemical binding, Nicotinamide might also have biological interference effects, as it is a PARP-1 inhibitor and an NAD^+^ precursor, which may influence cellular pathways related to DNA damage response; however, this mechanism remains hypothetical and was not directly investigated in this study.

These results indicate that Nicotinamide can modulate Cisplatin cytotoxicity in lung cancer cellular in vitro models (A549, PC-9), particularly under specific exposure conditions. However, given the supra-physiological concentrations used in this study, the direct clinical relevance of these observations remains uncertain. Since the current studies are based on in vitro models, further in vivo studies are necessary to assess their potential significance in clinical settings. Future studies should incorporate direct analytical approaches (e.g., LC-MS-based speciation and DNA adduct quantification) as well as investigations under clinically relevant conditions to better define the significance of these interactions. Consequently, while the present exploratory multi-tiered study provides valuable insight into these interactions, full solution speciation by LC-MS/ESI-MS remains highly desirable in future studies.

Collectively, these findings highlight the importance of further investigation into potential drug–nutrient interactions in the context of Platinum-based therapies. Overall, this study provides a mechanistic framework for understanding Cisplatin–Nicotinamide interactions under controlled conditions, while emphasizing that translation to clinical settings requires careful validation.

## Figures and Tables

**Table 1:** Values of the change in Gibbs Free Energy of reaction (ΔG_r_) for the complexations of the first product of hydrolysis of Cisplatin (CisPt) with Nicotinamide (B3) and nucleobases (Adenine (A), Guanine (G)) at two levels of calculations, B3LYP/6-31G(d,p)/LANL2DZ and MN15/def2-TZV.

Reactions	ΔG_r_ [kcal/mol]	
	B3LYP/6-31G(d,p)/LANL2DZ	MN15/def2-TZVP
CisPt1 + B3 → CisPt1-B3	−26.09	−28.70
CisPt1+ A → CisPt1-A	−24.72	−29.34
CisPt1 + G → CisPt1-G	−27.07	−31.91

**Table 2:** The detailed comparison of theoretical and experimental results of UV-Vis study for the Cisplatin–Nicotinamide and nucleobases–Cisplatin complexes. The wavelength values (λ) given in parentheses are the values for the absorbance maxima of Adenine, Guanine, Nicotinamide, and Cisplatin taken from the literature data [66,67,68,69,70,71,72,73].

Compounds	Experimental	B3LYP/6-31G(d,p)/LANL2DZ		MN15/def2-TZVP	
	λ_max_ [nm]	λ_max_ [nm]	MAD	λ_max_ [nm]	MAD
Adenine (A)	218.0 (220.0) [66,67] 264.0 (260.0) [66,67]	237.2	9.6 (8.6) 13.4 (11.4)	234	8.0 (7.0) 15.0 (13.0)
Guanine (G)	219.0 (219.0) [66,67] (249.0) [66,67] 260.0 (270.0) [66,67]	232.5	6.8 (6.8) (8.3) 13.8 (18.8)	227.8	4.4 (4.4) (10.6) 16.1 (21.1)
Nicotinamide (B3)	220.0 (210.0) [68,69] (250.0) [68,69] 262.0 (260.0/262.0) [68,69]	246.4	13.2 (18.2) (1.8) 7.8 (6.8/7.8)	244.6	12.3 (17.3) (2.7) 8.7 (7.7/8.7)
Cisplatin (CisPt1) (*cis*-[Pt(NH_3_)_2_Cl]^+^, (CisPt1)) ^a^	218.0 (207.0) [71] 276.0 (260.0, 280.0) [72,73]	481.8	131.9 ^a^ (137.4) 102.9 ^a^ (110.9, 100.9)	463.7	122.9 ^a^ (128.4) 93.9 ^a^ (101.9, 91.9)
Complex CisPt1-A, Mixture of CisPt1 and A	218.0 265.0	316.6	49.3 25.8	287.4	34.7 11.2
Complex CisPt1-G, Mixture of CisPt1 and G	220.0 265.0	319.2	49.6 27.1	286.8	33.4 10.9
Complex CisPt1-B3, Mixture of CisPt1 and B3	218.0 266.0	312.8	47.4 23.4	289.0	35.5 11.5
*cis*-[Pt(NH_3_)_2_NicotinamideCl]NO_3_, synthesized complex	222.0 265.0	312.8	45.4 21.4	289.0	33.5 9.5
Average value			* 20.4		* 18.6
			** 52.4		** 39.7
			*** 34.6		*** 27.1

^a^ First product of hydrolysis of Cisplatin (*cis*-[Pt(NH_3_)_2_Cl]^+^, (CisPt1)) used during the theoretical study. * MAD values considering only the closest wavelength values from experimental and theoretical research, excluding Cisplatin. ** MAD values considering only the wavelength values for compounds containing Platinum from experimental and theoretical research. *** MAD values considering all wavelength values from experimental and theoretical research.

**Table 3:** Minimum inhibitory concentrations (MICs) of CisPt, B3, and the synthesized complex.

Strain	CisPt [µM]	B3 [µM]	Complex [µM]
ECO ATCC 25922	450	>3600	>3600/3600
ECO 1 (ESBL+)	275	>3600	>3600/3600
SAU ATCC 29213	3600	>3600	>3600/3600
SAU1 (MRSA)	3600	>3600	>3600/3600
EFA ATCC 29212	900	>3600	3600
EFA1 (VRE)	1800	>3600	3600
PAE ATCC 27853	113	>3600	1800
PAE 1 (VIM-2)	113	>3600	1800
KPN ATCC 700603	900	>3600	3600
KPN 1 (OXA-48)	450	>3600	3600
BSU ATCC 7972	1800	>3600	1800
BSU 1	1800	>3600	1800
CAL ATCC 90028	1800	>3600	3600
CAL 1 (fluconazole-resistant)	1800	>3600	3600

**Table 4:** Minimum inhibitory concentrations (MICs) of CisPt + B3 mixture.

Strain	CisPt + B3 [µM] (CisPt/B3)
ECO ATCC 25922	1800/3600
ECO 1 (ESBL+)	900/1800
SAU ATCC 29213	3600/7200
SAU1 (MRSA)	3600/7200
EFA ATCC 29212	1800/3600
EFA1 (VRE)	3600/7200
PAE ATCC 27853	57/113
PAE 1 (VIM-2)	225/450
KPN ATCC 700603	450/900
KPN 1 (OXA-48)	1800/3600
BSU ATCC 7972	1800/3600
BSU 1	1800/3600
CAL ATCC 90028	900/1800
CAL 1 (fluconazole-resistant)	900/1800

**Table 5:** Qualitative assessment of interactions between CisPt and vitamin B3 in the mixture.

Strain	MIC CisPt Alone [µM]	MIC CisPt + B3 [µM] (CisPt Component)	Type of Interaction
ECO ATCC 25922	450	1800	Antagonism
ECO 1 (ESBL+)	275	900	Antagonism
SAU ATCC 29213	3600	3600	Indifference
SAU1 (MRSA)	3600	3600	Indifference
EFA ATCC 29212	900	1800	Antagonism
EFA1 (VRE)	1800	3600	Antagonism
PAE ATCC 27853	113	57	Synergism
PAE 1 (VIM-2)	113	225	Antagonism
KPN ATCC 700603	900	450	Synergism
KPN 1 (OXA-48)	450	1800	Antagonism
BSU ATCC 7972	1800	1800	Indifference
BSU 1	1800	1800	Indifference
CAL ATCC 90028	1800	900	Synergism
CAL 1 (fluconazole-resistant)	1800	900	Synergism

**Table 6:** The IC_50_ values of Cisplatin (CisPt), CisPt1-B3 complex, and the freshly prepared mixture of CisPt and B3 (CisPt+B3 Mix) in the A549 cell line. For both Cisplatin and the synthesized complex, the tested concentration range was 0.04–125 µM. In the freshly prepared mixture (CisPt+B3 Mix), Cisplatin was maintained at a constant 1:2 molar ratio relative to Nicotinamide across the same concentration range (0.04–125 µM of CisPt and 0.08–250 µM of B3).

Time Point	CisPt	CisPt-B3 Complex	CisPt+B3 Mix (1:2)
**24 h**	50.86 ± 11.92 µM	>125 µM (1307.9 ± 485.6)	60.43 ± 10.59 µM
**48 h**	8.612 ± 1.402 µM	>125 µM (255.2 ± 35.23)	8.783 ± 1.103 µM
**72 h**	5.327 ± 0.733 µM	>125 µM (284.3 ± 41.73)	5.733 ± 0.703 µM

**Table 7:** The IC_50_ values of Cisplatin (CisPt), CisPt1-B3 complex, and the freshly prepared mixture of CisPt and B3 (CisPt+B3 Mix) in the PC-9 cell line. For both Cisplatin and the synthesized complex, the tested concentration range was 0.04–125 µM. In the freshly prepared mixture (CisPt+B3 Mix), Cisplatin was maintained at a constant 1:2 molar ratio relative to Nicotinamide across the same concentration range (0.04–125 µM of CisPt and 0.08–250 µM of B3).

Time Point	CisPt	CisPt-B3 Complex	CisPt+B3 Mix (1:2)
**24 h**	10.38 ± 1.875 µM	>125 µM (365.4 ± 70.71)	9.157 ± 1.91 µM
**48 h**	2.107 ± 0.213 µM	48.620 ± 7.404 µM	1.401 ± 0.128 µM
**72 h**	0.990 ± 0.105 µM	25.22 ± 3.370 µM	0.809 ± 0.057 µM

## Data Availability

Requests for further information and resources should be directed to and will be fulfilled by the lead contact, Beata Szefler (beatas@cm.umk.pl). This study did not generate new unique reagents.

## Supplementary Materials

The following supporting information can be downloaded at: https://www.mdpi.com/article/10.3390/ijms27114989/s1.

## List of Abbreviations Used

CisPt	Cisplatin, *cis*-[Pt(NH_3_)_2_Cl_2_]
CDDP	Cisplatin, *cis*-[Pt(NH_3_)_2_Cl_2_], name used in the experiment part with cell lines
CisPt1	Mono-aquated form of Platinum (*cis*-[Pt(NH_3_)_2_Cl]^+^ formed after the separation of a water molecule (H_2_O) from the first hydrolysis product of Cisplatin *cis*-[Pt(NH_3_)_2_Cl(H_2_O)]^+^
*cis*-[Pt(NH_3_)_2_Cl(H_2_O)]^+^	First product of hydrolysis of Cisplatin (*cis*-[Pt(NH_3_)_2_Cl_2_])
*cis*-[Pt(NH_3_)_2_NicotinamideCl]NO_3_	Synthesized study complex
B3	Vitamin B3
A	Adenine
G	Guanine
NAD^+^	Nicotinamide Adenine Dinucleotide
ROS	Reactive Oxygen Species
PARP-1	Poly-ADP-ribose polymerase-1
CisPt1-B3 complex	*cis*-[Pt(NH_3_)_2_Cl(Nicotinamide)]^+^ complex; complex of mono-aquated form of Platinum (*cis*-[Pt(NH_3_)_2_Cl]^+^ with vitamin B3
CisPt1-A complex	*cis*-[Pt(NH_3_)_2_Cl(Adenine)]^+^ complex; complex of mono-aquated form of Platinum (*cis*-[Pt(NH_3_)_2_Cl]^+^ with Adenine
CisPt1-G complex	*cis*-[Pt(NH_3_)_2_Cl(Guanine)]^+^ complex; complex of mono-aquated form of Platinum (*cis*-[Pt(NH_3_)_2_Cl]^+^ with Guanine
NMR	Nuclear Magnetic Resonance spectroscopy
DSC	Differential scanning calorimetry
ΔG_r_	Gibbs Free Energy of reaction
DFT	Density functional theory
B3LYP/6-31G(d,p)/LANL2DZ	Hybrid functional B3LYP with a 6-31G(d,p) basis set for non-metals and LANL2DZ for Platinum
MN15/def2-TZV	Minnesota global hybrid meta-GGA functional (MN15) with the def2-TZVP basis set
PBE0	Hybrid functional of Perdew, Burke, and Ernzerhof used for UV-Vis spectral calculations
PCM	Polarizable Continuum Model (specifically IEF-PCM used to simulate solvent effects)
ZPE	Zero Point Energies
UV-Vis	Ultraviolet-Visible spectroscopy
HOMO	Highest Occupied Molecular Orbital
LUMO	Lowest Unoccupied Molecular Orbital
ΔE_gap_	Energy gap between LUMO and HOMO
χ	Absolute electronegativity
μ	Chemical potential
η	Absolute hardness
σ	Absolute softness
σ	Global electrophilicity
S	Global softness
ΔN_max_	Maximum additional electronic charge
MAD	Mean Absolute Deviation
λ_max_	Wavelength of maximum absorption
MIC	Minimum inhibitory concentration
AMPs	Antimicrobial peptides
MDR	Multi-drug resistant
ESBL+	Extended-spectrum beta-lactamase-producing bacteria
MRSA	Methicillin-resistant Staphylococcus aureus
VRE	Vancomycin-resistant Enterococcus
FICI	Fractional inhibitory concentration index
ECO	*Escherichia coli*
SAU	*Staphylococcus aureus*
EFA	*Enterococcus faecalis*
PAE	*Pseudomonas aeruginosa*
KPN	*Klebsiella pneumoniae*
BSU	*Bacillus subtilis*
CAL	*Candida albicans*
NSCLC	Non-small cell lung cancer
A549	Human non-small cell lung cancer (adenocarcinoma) cell lin
PC–9	Human non-small cell lung cancer cell line
IC50	Half-maximal inhibitory concentration (concentration of a drug that is required for 50% inhibition in vitro)
MTT	3-(4,5-dimethylthiazol-2-yl)-2,5-diphenyltetrazolium bromide (colorimetric assay for assessing cell metabolic activity/viability)
