# Supplementary material for: Molecular and Functional Interactions Between Cisplatin and Nicotinamide: A Combined Computational, Spectroscopic, and Biological Study
Source: Int J Mol Sci. 2026 May 30;27(11):4989. doi: 10.3390/ijms27114989 (PMC13257153; doi:10.3390/ijms27114989)
Supplement: Supplementary file 1 [file ijms-27-04989-s001.zip › ijms-4179886-supplementary.pdf]

Molecular and Functional Interactions Between Cisplatin and Nicotinamide: A Combined Computational, Spectroscopic, and Biological Study

Beata Szefler [1,*], Magdalena Wujak [2], Agnieszka Skotnicka [3], Krzysztof Skowron [4], Julia Czuba [4], Przemysław Czeleń [1], Kamil Szupryczyński [5] and Piotr Cysewski [1]

[1] Department of Physical Chemistry, Faculty of Pharmacy, Collegium Medicum, Nicolaus Copernicus University, Kurpińskiego 5, 85-096 Bydgoszcz, Poland
[2] Department of Medicinal Chemistry, Faculty of Pharmacy, Collegium Medicum in Bydgoszcz, Nicolaus Copernicus University, Jurasza 2, 85-089 Bydgoszcz, Poland
[3] Faculty of Chemical Technology and Engineering, Bydgoszcz University of Science and Technology, Seminaryjna 3, 85-326 Bydgoszcz, Poland
[4] Department of Microbiology, Faculty of Pharmacy, Collegium Medicum in Bydgoszcz, Nicolaus Copernicus University, M. Curie Skłodowskiej 9, 85-094 Bydgoszcz, Poland
[5] Faculty of Pharmacy, Doctoral School of Medical and Health Sciences, Collegium Medicum, Nicolaus Copernicus University, Jagiellońska 13, 85-067 Bydgoszcz, Poland
* Correspondence: beatas@cm.umk.pl

Table of contents:

Figure S1. Structure of study compounds.

Figure S2. ^{1}H NMR spectra of *cis*-[Pt(NH_3)$_2$NicotinamideCl]NO_3 complex in DMSO-*d$_6$*.

Figure S3. ^{13}C NMR spectra of *cis*-[Pt(NH_3)$_2$NicotinamideCl]NO_3 complex in DMSO-*d$_6$*.

Figure S4. Thermogram of differential scanning calorimetry (DSC) of *cis*-[Pt(NH_3)$_2$nicotinamideCl]NO_3 complex.

Figure S5. The FT IR spectrum of *cis*-[Pt(NH_3)$_2$NicotinamideCl]NO_3 complex.

Figure S6. Experimental UV-Vis absorbance spectrum of nucleobases (Adenine, Guanine), Nicotinamide and Cisplatin during incubation at 37 °C in phosphate buffer at pH 7.4.

Figure S7. Computed UV-Vis spectra for selected nucleobases Adenine (A) and Guanine (G) and their complexes with the first product of hydrolysis of Cisplatin (CisPt1), CisPt1-A, CisPt1-G. The calculations were performed after optimization at the B3LYP/6-31G(d,p) LANL2DZ and MN15/def2-TZV levels of theory, using PCM with water solvent. All spectroscopic calculations utilized the PBE0 functional.

Figure S8. Computed UV-Vis spectra for Nicotinamide (B3), the first product of hydrolysis of Cisplatin (CisPt1) and CisPt1-B3 complex. The calculations were performed after optimization at the B3LYP/6-31G(d,p)/LANL2DZ and MN15/def2-TZV levels of theory, using PCM with water solvent. All spectroscopic calculations utilized the PBE0 functional.

Table S1. HOMO LUMO (in eV) values and the parameters that indicate the chemical behaviour of the studied molecules, such as energy gap (ΔE_{gap}), absolute electronegativity (χ), chemical potentials (μ), absolute hardness (η), absolute softness (σ), global electrophilicity (ω), global softness (S) and additional electronic charge (ΔN_{max}) at two levels of calculations, B3LYP/6-31G(d,p)/LANL2DZ and MN15/def2-TZV.

Figure S9. HOMO LUMO plots of nucleobases Adenine (A) and Guanine (G) and their complexes with the first product of hydrolysis of Cisplatin (CisPt1) (isovalue=0.02, density=0.0004).

Figure S10. HOMO LUMO plots of Nicotinamide (B3), the first product of hydrolysis of Cisplatin (CisPt1) and CisPt1-B3 complex (isovalue=0.02, density=0.0004).

Figure S11. Effects of Cisplatin, synthesized complex, *cis*-[Pt$(NH_3)_2$NicotinamideCl]NO_3, and fresh mixture of B3 and CisPt (2:1) on the viability of A549 cell line, assessed using the MTT assay.

Figure S12. Effects of Cisplatin, synthesized complex, *cis*-[Pt$(NH_3)_2$NicotinamideCl]NO_3, and fresh mixture of B3 and CisPt (2:1) on the viability of PC-9 cell line, as assessed using the MTT assay.

Figure S13. Effect of vitamin B3 supplementation on the viability of A549 (A) and PC-9 (B), assessed using the MTT assay. The cells were sequentially treated with vitamin B3 at indicated concentrations every 24 h for 72 h in total.

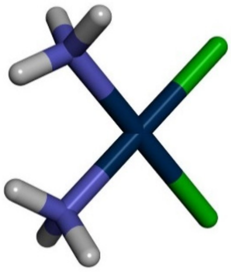

Cisplatin (CisPt, CDDP)

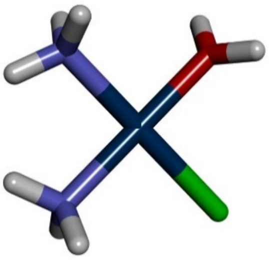

First-hydrolysis product of Cisplatin (CisPt1)

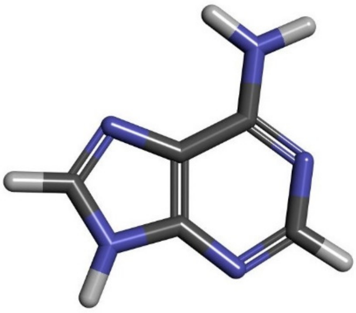

Adenine (A)

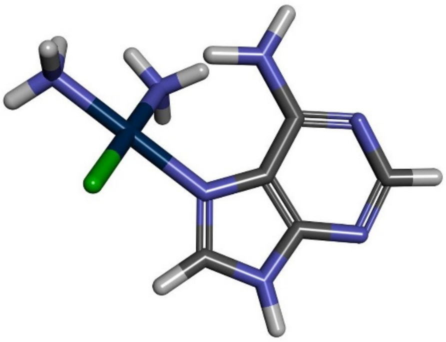

Complex of Cisplatin (CisPt1)–Adenine (A)

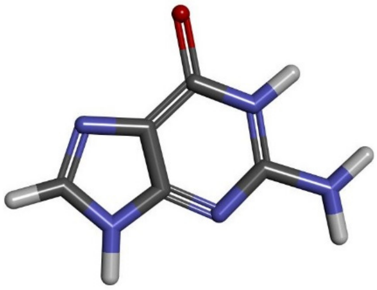

Guanine (G)

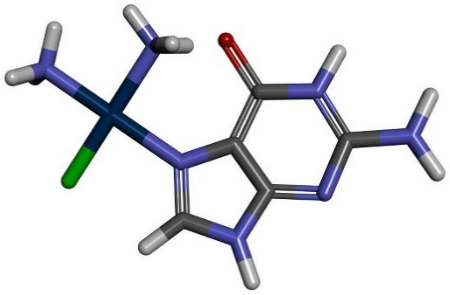

Complex of Cisplatin (CisPt1)–Guanine (G)

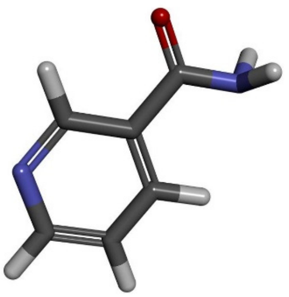

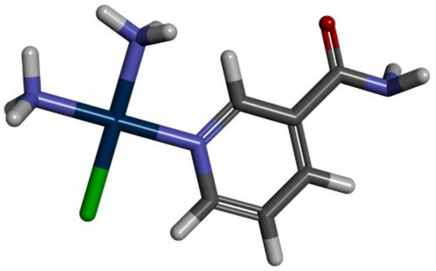

Nicotinamide (B3) Complex of Cisplatin (CisPt1)–Nicotinamide (B3)

Figure S1. Structure of study compounds.

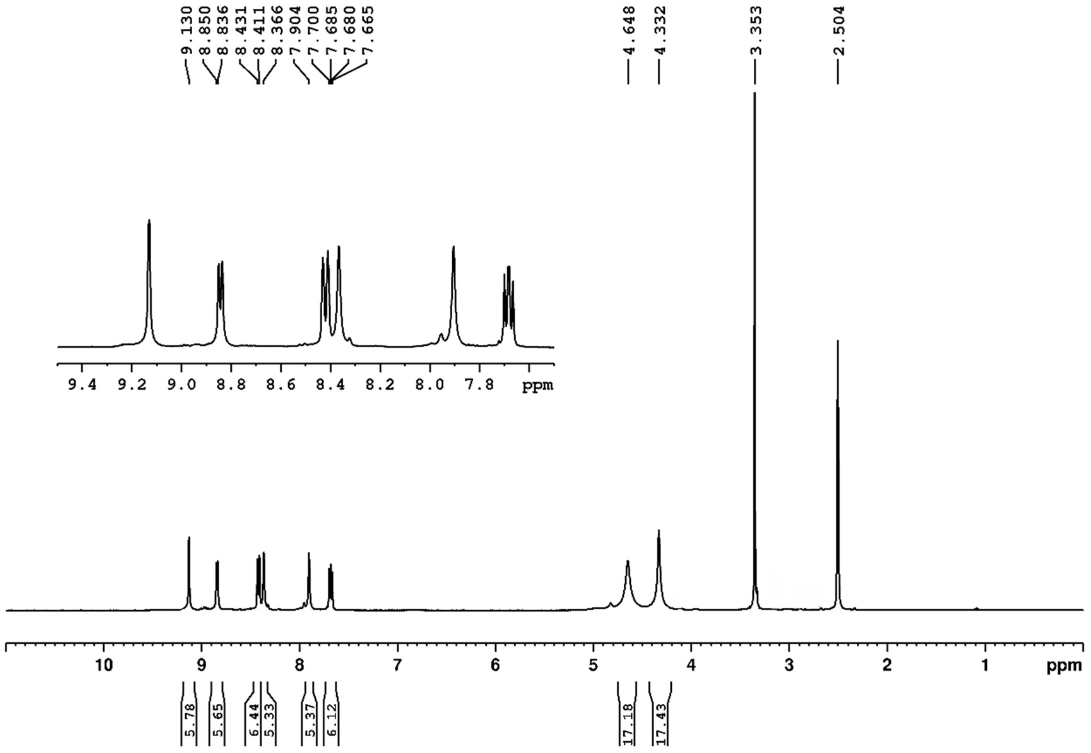

Figure S2. ^{1}H NMR spectra of *cis*-[Pt(NH_3)$_2$NicotinamideCl]NO_3 complex in DMSO-d_6.

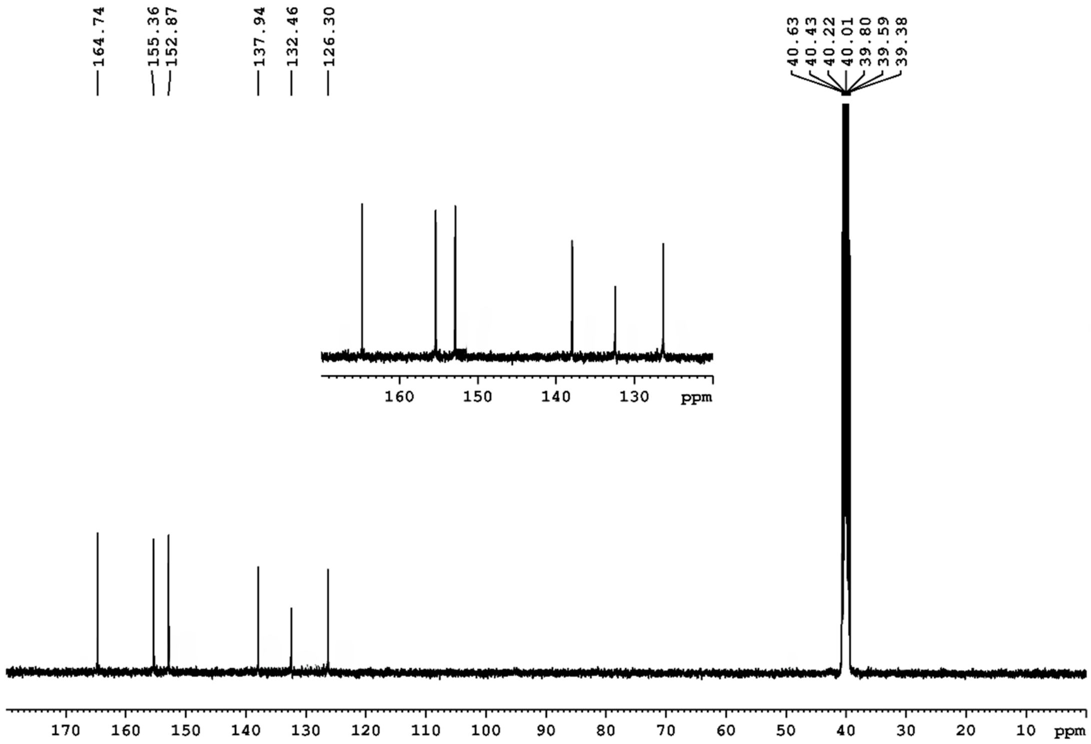

Figure S3. ^{13}C NMR spectra of *cis*-[Pt$(NH_3)_2$NicotinamideCl]NO_3 complex in DMSO-d_6.

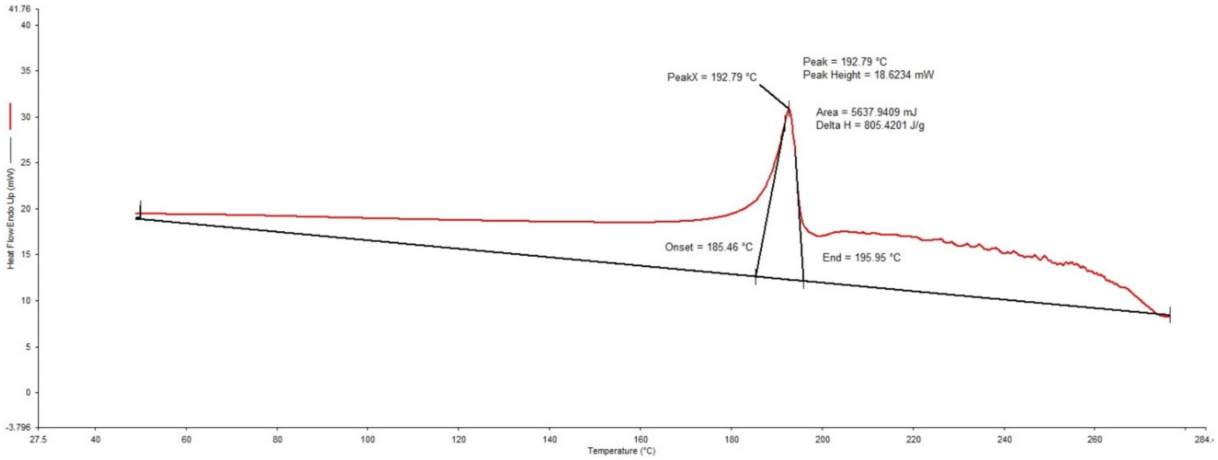

Figure S4. Thermogram of differential scanning calorimetry (DSC) of *cis*-[Pt$(NH_3)_2$NicotinamideCl]NO_3 complex.

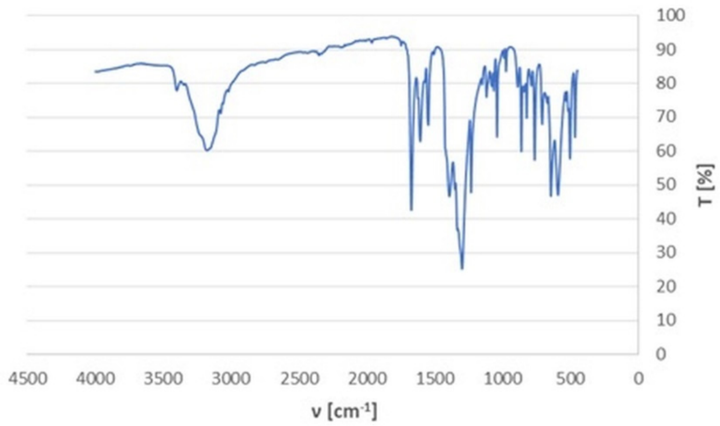

Figure S5. The FT IR spectrum of *cis*-[Pt(NH_3)$_2$NicotinamideCl]NO_3 complex.

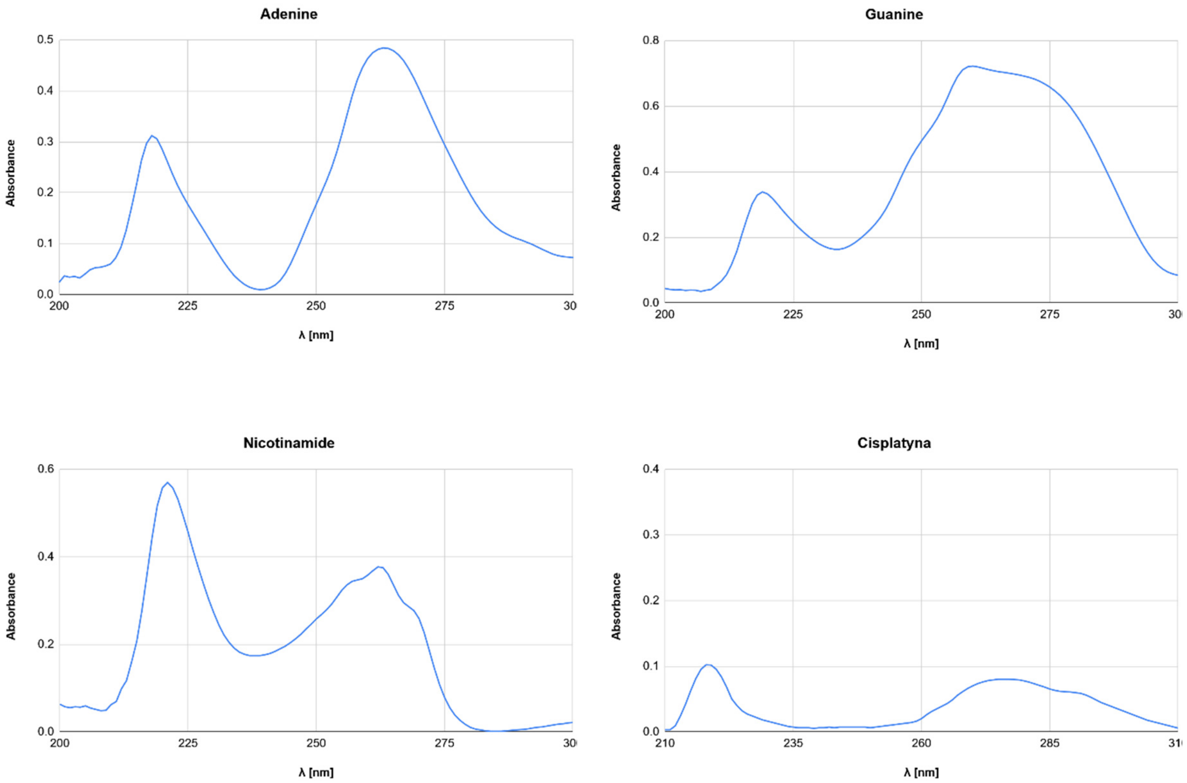

Figure S6. Experimental UV-Vis absorbance spectrum of nucleobases (Adenine (A), Guanine (G)), Nicotinamide (B3) and Cisplatin (CisPt) during incubation at 37 °C in phosphate buffer at pH 7.4. Cisplatin was present at a concentration of 32.7 µM, while the nucleobases and Nicotinamide were present at 65.3 µM, corresponding to a 1:2 molar ratio.

Methods	
B3LYP/6-31G(d,p)/LANL2DZ	MN15/def2-TZVP

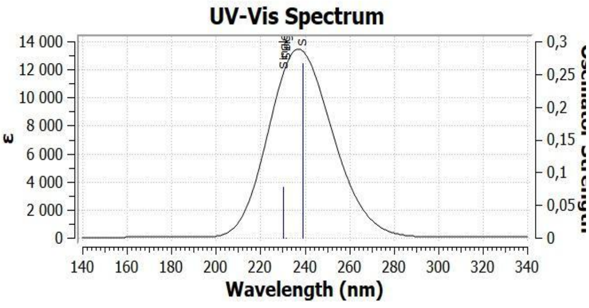

Adenine (A)

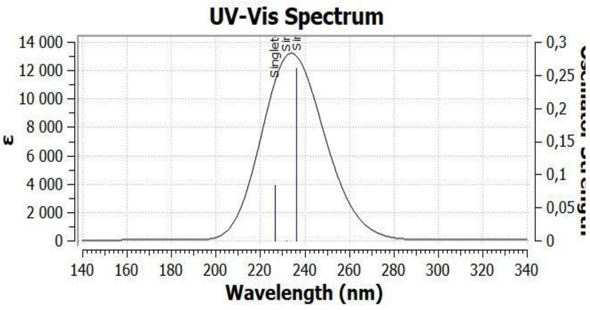

Adenine (A)

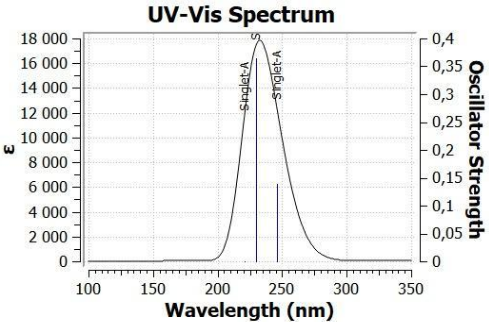

Guanine (G)

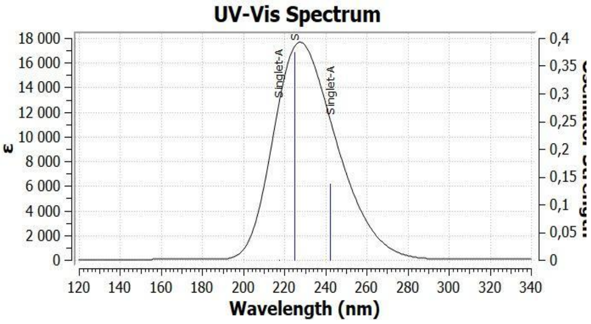

Guanine (G)

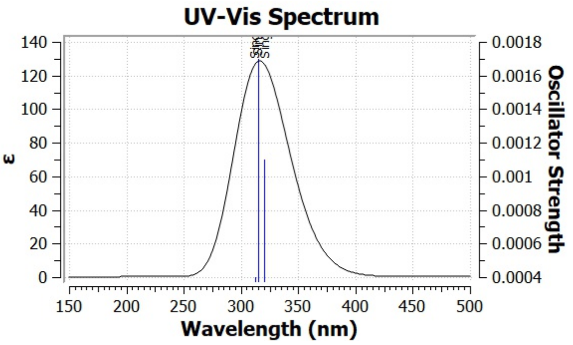

Complex CisPt1-A

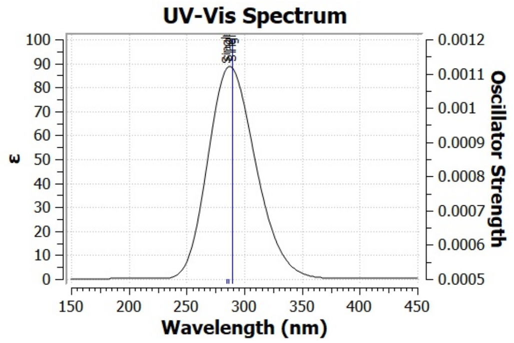

Complex CisPt1-A

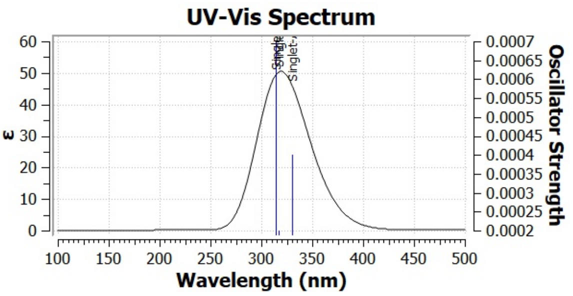

Complex CisPt1-G

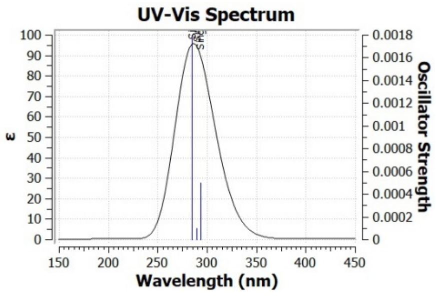

Complex CisPt1-G

Figure S7. Computed UV-Vis spectra for selected nucleobases Adenine (A) and Guanine (G) and their complexes with the first product of hydrolysis of Cisplatin (CisPt1), CisPt1-A, CisPt1-G. The calculations were performed after optimization at the B3LYP/6-31G(d,p) LANL2DZ and MN15/def2-TZV levels of theory, using PCM with water solvent. All spectroscopic calculations utilized the PBE0 functional.

<table>
<tr><th colspan="2">Methods</th></tr>
<tr><th>B3LYP/6-31G(d,p)/LANL2DZ</th><th>MN15/def2-TZVP</th></tr>
<tr><td>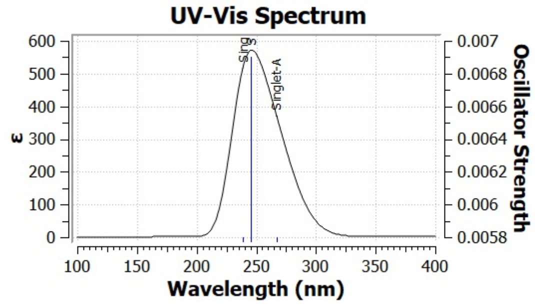

Nicotinamide (B3)</td><td>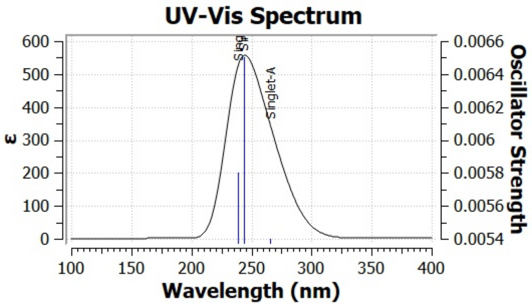

Nicotinamide (B3)</td></tr>
<tr><td>Complex CisPt1-B3
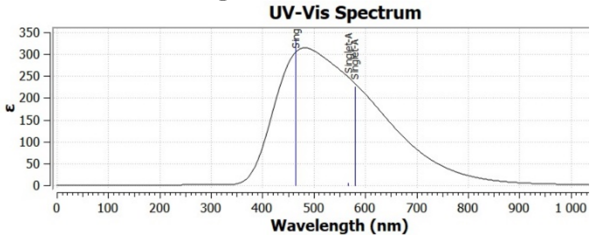

CisPt1</td><td>Complex CisPt1-B3
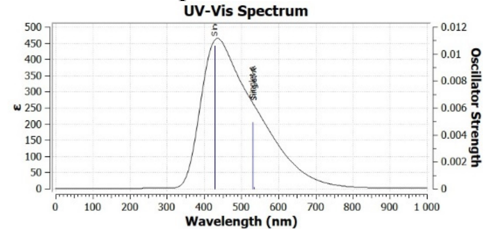

CisPt1</td></tr>
</table>

Figure S8. Computed UV-Vis spectra for Nicotinamide (B3), the first product of hydrolysis of Cisplatin (CisPt1) and CisPt1-B3 complex. The calculations were performed after optimization at the B3LYP/6-31G(d,p)/LANL2DZ and MN15/def2-TZV levels of theory, using PCM with water solvent. All spectroscopic calculations utilized the PBE0 functional.

Table S1. HOMO LUMO (in eV) values and the parameters that indicate the chemical behaviour of the studied molecules, such as energy gap (ΔE_{gap}), absolute electronegativity (χ), chemical potentials (μ), absolute hardness (η), absolute softness (σ), global electrophilicity (ω), global softness (S) and additional electronic charge (ΔN_{max}) at two levels of calculations, B3LYP/6-31G(d,p)/LANL2DZ and MN15/def2-TZV.

	**B3LYP/6-31G(d,p)/LANL2DZ**											
	**HOMO (eV)**	**LUMO (eV)**	**E. gap (eV)**	**η (eV)**	**μ**	**σ**	**s**	**χ**	**ω**	**ΔN_{max}**	**ΔE_n**	**Δ_{ee}**
Adenine (A)	-6.3	-0.5	5.8	2.9	3.4	0.3	0.2	3.4	1.9	-1.2	17.0	10.3
Guanine (G)	-6.0	-0.1	5.9	2.9	3.1	0.3	0.2	3.1	1.6	-1.0	13.8	7.7
Nicotinamide (B3)	-7.3	-1.2	6.2	3.1	4.3	0.3	0.2	4.3	3.0	-1.4	29.3	20.7
CisPt1	-7.5	-3.2	4.3	2.1	5.4	0.5	0.2	5.4	6.8	-2.5	34.4	23.6
Complex CisPt1-A	-6.7	-1.3	5.3	2.7	4.0	0.4	0.2	4.0	3.0	-1.5	22.6	14.6
Complex CisPt1-G	-6.4	-1.2	5.2	2.6	3.8	0.4	0.2	3.8	2.8	-1.5	20.3	12.7
Complex CisPt1-B3	-7.3	-1.8	5.5	2.7	4.5	0.4	0.2	4.5	3.7	-1.6	29.8	20.8

	**MN15/def2-TZV**											
	**HOMO (eV)**	**LUMO (eV)**	**E. gap (eV)**	**η (eV)**	**μ**	**σ**	**s**	**χ**	**ω**	**ΔN_{max}**	**ΔE_n**	**Δ_{ee}**
Adenine (A)	-6.3	-0.4	5.9	3.0	3.3	0.3	0.2	3.3	1.9	-1.1	16.9	10.2
Guanine (G)	-6.0	0.0	5.9	3.0	3.0	0.3	0.2	3.0	1.5	-1.0	13.2	7.3
Nicotinamide (B3)	-7.3	-1.1	6.2	3.1	4.2	0.3	0.2	4.2	2.9	-1.4	28.8	20.4
CisPt1	-7.5	-3.0	4.4	2.2	5.3	0.5	0.2	5.3	6.3	-2.4	33.5	23.0
Complex CisPt1-A	-6.6	-0.9	5.8	2.9	3.8	0.3	0.2	3.8	2.5	-1.3	21.2	13.7
Complex CisPt1-G	-6.4	-0.8	5.6	2.8	3.6	0.4	0.2	3.6	2.3	-1.3	18.5	11.4
Complex CisPt1-B3	-7.2	-1.7	5.5	2.7	4.4	0.4	0.2	4.4	3.6	-1.6	28.5	19.7

**Methods**			
**B3LYP/6-31G(d,p)/ LANL2DZ**		**MN15/def2-TZVP**	
**HOMO**	**LUMO**	**HOMO**	**LUMO**
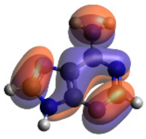	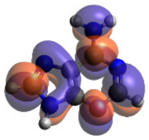	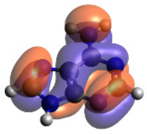	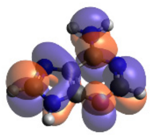
Adenine (A)		Adenine (A)	

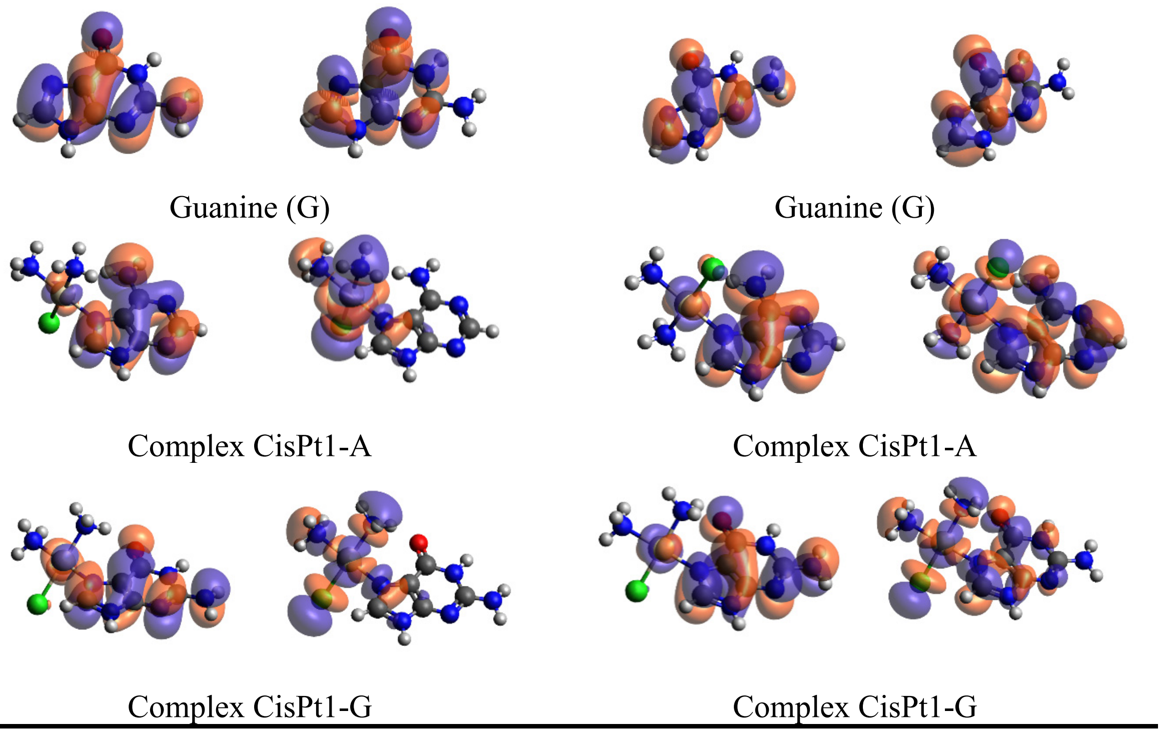

Figure S9. HOMO LUMO plots of nucleobases Adenine (A) and Guanine (G) and their complexes with the first product of hydrolysis of Cisplatin (CisPt1) (isovalue = 0.02, density = 0.0004).

**Methods**			
**B3LYP/6-31G(d,p)/ LANL2DZ**		**MN15/def2-TZVP**	
**HOMO**	**LUMO**	**HOMO**	**LUMO**

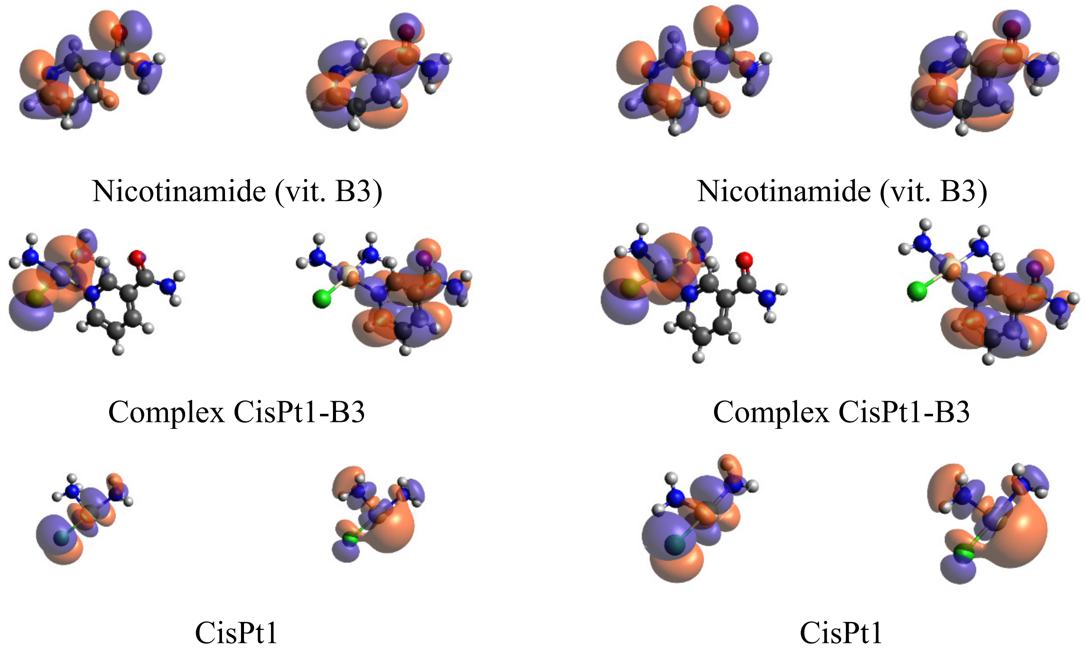

Figure S10. HOMO LUMO plots of Nicotinamide (B3), the first product of hydrolysis of Cisplatin (CisPt1) and CisPt1-B3 complex (isovalue = 0.02, density = 0.0004).

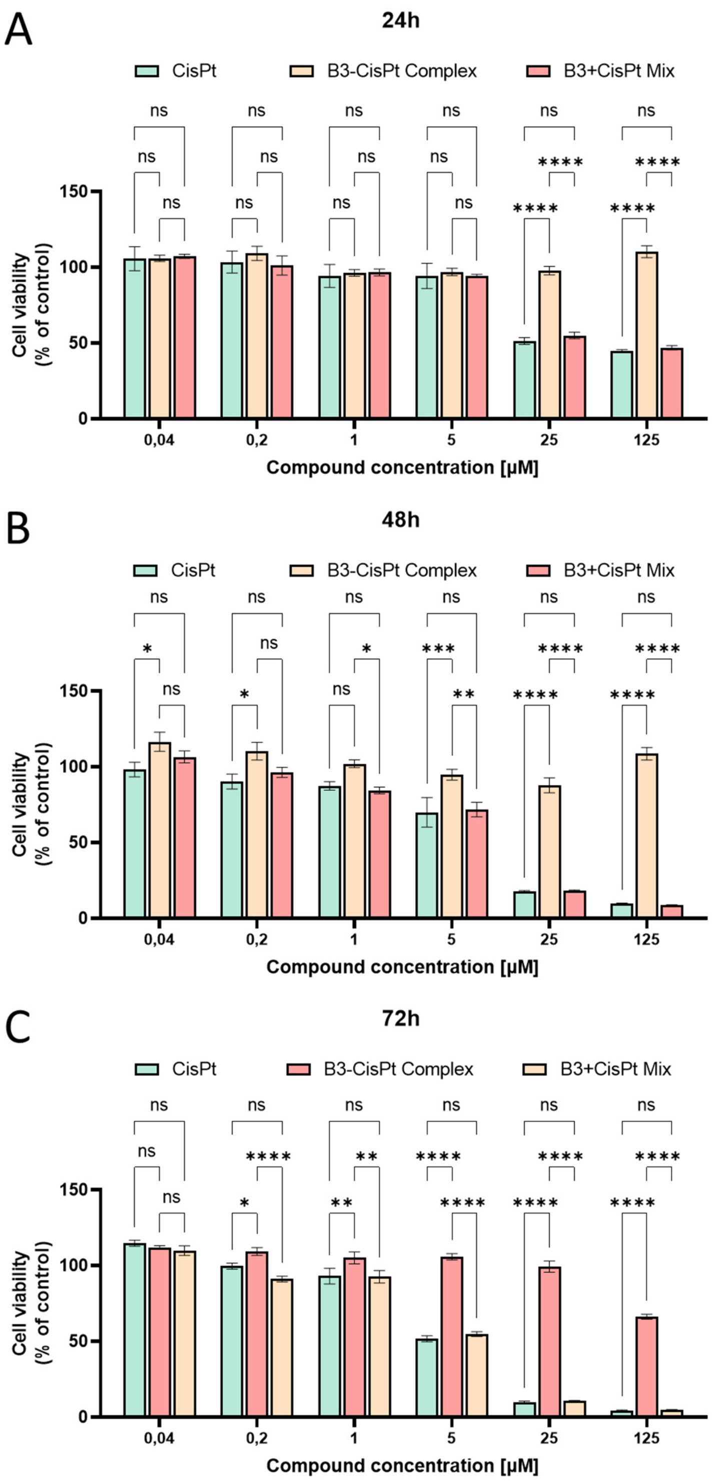

Figure S11. Effects of Cisplatin, synthesized complex, *cis*-[Pt(NH_3)$_2$NicotinamideCl]NO_3, and fresh mixture of B3 and CisPt (2:1) on the viability of A549 cell line, assessed using the MTT assay.

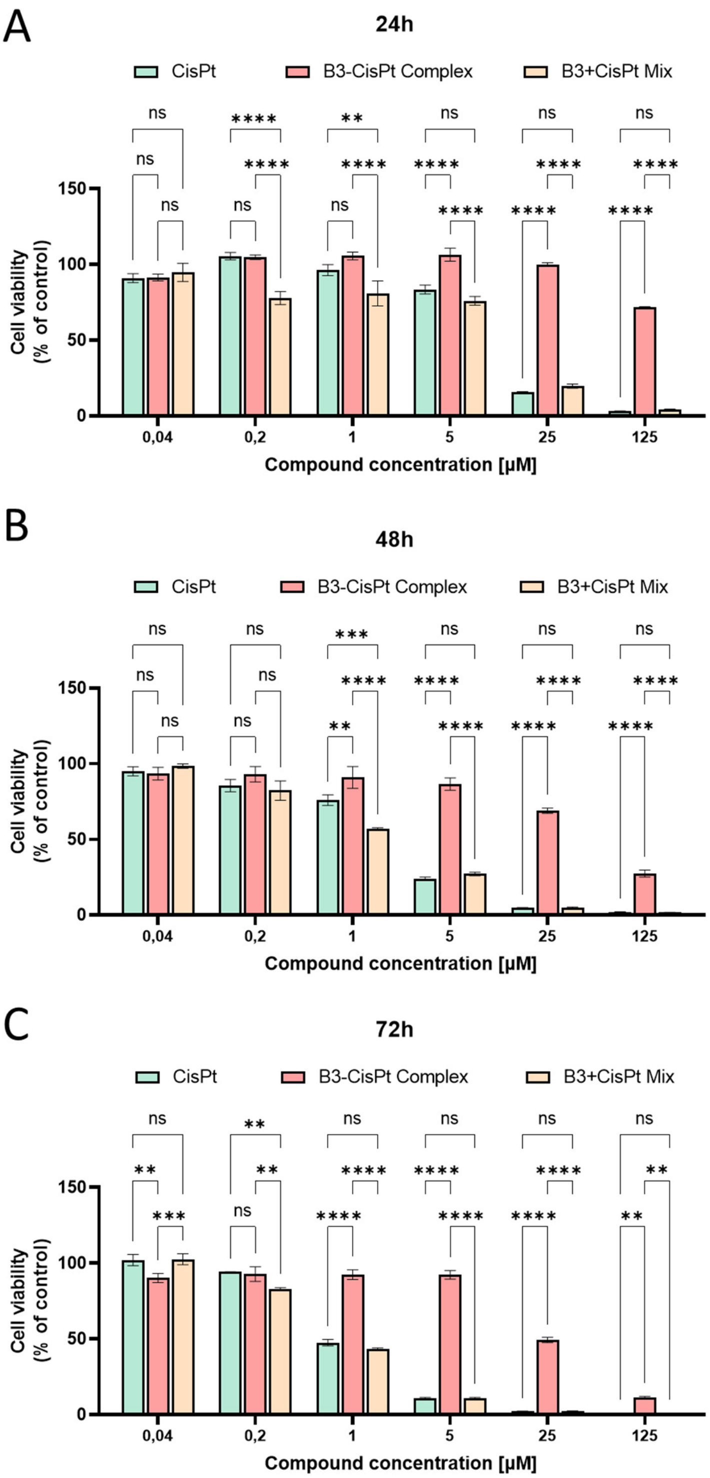

Figure S12. Effects of Cisplatin, synthesized complex, *cis*-$[Pt(NH_3)_2NicotinamideCl]NO_3$, and fresh mixture of B3 and CisPt (2:1) on the viability of PC-9 cell line, as assessed using the MTT assay.

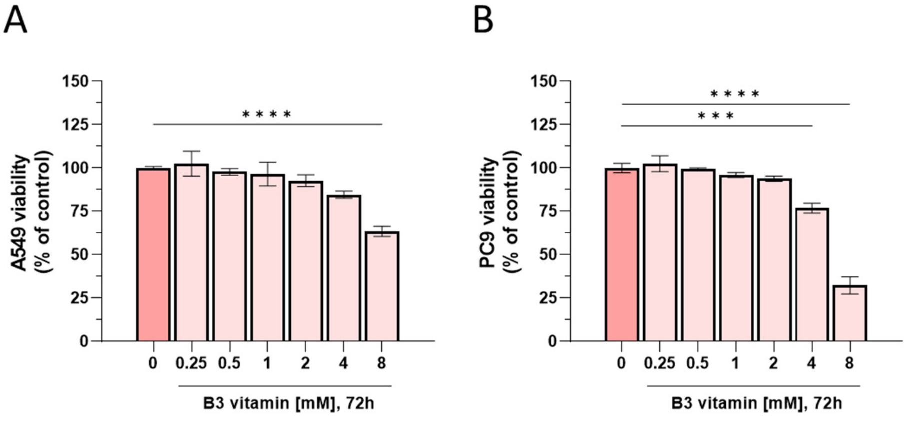

Figure S13 Effect of vitamin B3 supplementation on the viability of A549 (A) and PC-9 (B), assessed using the MTT assay. The cells were sequentially treated with vitamin B3 at indicated concentrations every 24 h for 72 h in total.

List of Abbreviations Used:

CisPt → Cisplatin, *cis*-$[Pt(NH_3)_2Cl_2]$

CDDP → Cisplatin, *cis*-$[Pt(NH_3)_2Cl_2]$, name used in the experiment part with cell lines

CisPt1 → mono-aquated form of Platinum (*cis*-$[Pt(NH_3)_2Cl]^+$ formed after the separation of a water molecule (H_2O) from the first hydrolysis product of Cisplatin *cis*-$[Pt(NH_3)_2Cl(H_2O)]^+$

cis-$[Pt(NH_3)_2Cl(H_2O)]^+$ → first product of hydrolysis of Cisplatin (*cis*-$[Pt(NH_3)_2Cl_2]$)

cis-$[Pt(NH_3)_2$NicotinamideCl$]NO_3$ → synthesized study complex

B3 → Vitamin B3

A → Adenine

G → Guanine

NAD^+ → Nicotinamide Adenine Dinucleotide

ROS → Reactive Oxygen Species

PARP-1 → Poly-ADP-ribose polymerase-1

CisPt1-B3 complex → *cis*-$[Pt(NH_3)_2Cl$(Nicotinamide)$]^+$ complex; complex of mono-aquated form of Platinum (*cis*-$[Pt(NH_3)_2Cl]^+$ with Vitamin B3

CisPt1-A complex → *cis*-$[Pt(NH_3)_2Cl$(Adenine)$]^+$ complex; complex of mono-aquated form of Platinum (*cis*-$[Pt(NH_3)_2Cl]^+$ with Adenine

CisPt1-G complex → *cis*-$[Pt(NH_3)_2Cl$(Guanine)$]^+$ complex; complex of mono-aquated form of Platinum (*cis*-$[Pt(NH_3)_2Cl]^+$ with Guanine

NMR→ Nuclear Magnetic Resonance spectroscopy

DSC → Differential Scanning Calorimetry

ΔG_r → Gibbs Free Energy of reaction

DFT→ Density Functional Theory

B3LYP/6-31G(d,p)/LANL2DZ → hybrid functional B3LYP with a 6-31G(d,p) basis set for non-metals and LANL2DZ for Platinum

MN15/def2-TZV → Minnesota global hybrid meta-GGA functional (MN15) with the def2-TZVP basis set

PBE0 → hybrid functional of Perdew, Burke, and Ernzerhof used for UV-Vis spectral calculations

PCM → Polarizable Continuum Model (specifically IEF-PCM used to simulate solvent effects)

ZPE → Zero Point Energies

UV-Vis → Ultraviolet-Visible spectroscopy

HOMO → Highest Occupied Molecular Orbital

LUMO → Lowest Unoccupied Molecular Orbital

ΔE_{gap} → energy gap between LUMO and HOMO

χ → absolute electronegativity

μ → chemical potential

η → absolute hardness

σ → absolute softness

σ → global electrophilicity

S → global Softness

ΔN_{max} → maximum additional electronic charge

MAD → Mean Absolute Deviation

λ_{max} → wavelength of maximum absorption

MIC → Minimum Inhibitory Concentrations

AMPs → Antimicrobial Peptides

MDR → Multi-Drug Resistant

ESBL+ → Extended-Spectrum Beta-Lactamase-producing bacteria

MRSA → Methicillin-Resistant Staphylococcus aureus

VRE → Vancomycin-Resistant Enterococcus

FICI → Fractional Inhibitory Concentration Index

ECO → *Escherichia coli*

SAU → *Staphylococcus aureus*

EFA → *Enterococcus faecalis*

PAE → *Pseudomonas aeruginosa*

KPN → *Klebsiella pneumoniae*

BSU → *Bacillus subtilis*

CAL → *Candida albicans*

NSCLC → Non-Small Cell Lung Cancer

A549 → human non-small cell lung cancer (adenocarcinoma) cell lin

PC–9 → human non-small cell lung cancer cell line

IC50 → half-maximal inhibitory concentration (concentration of a drug that is required for 50% inhibition in vitro)

MTT → 3-(4,5-dimethylthiazol-2-yl)-2,5-diphenyltetrazolium bromide (colorimetric assay for assessing cell metabolic activity/viability)
